# Redox-sensitive δ^65^Cu isotopic fractionation in the tissue of the scleractinian coral *Stylophora pistillata:* a biomarker of holobiont photophysiology following volcanic ash exposure

**DOI:** 10.1093/mtomcs/mfaf011

**Published:** 2025-04-23

**Authors:** Frank Förster, Lucie Sauzéat, Christine Ferrier-Pagès, Stéphanie Reynaud, Tom E Sheldrake

**Affiliations:** Geovolco Team, Department of Earth Sciences, University of Geneva, Genève, Switzerland; Laboratoire Magmas et Volcans (LMV), Université Clermont Auvergne, CNRS, IRD, OPGC, F-63000 Clermont-Ferrand, France; Institut de Génétique, Reproduction et Développement (iGReD), Université Clermont Auvergne, CNRS, INSERM, F-63000 Clermont-Ferrand, France; Ecophysiology Team, Centre Scientifique de Monaco, Monaco, Monaco; Ecophysiology Team, Centre Scientifique de Monaco, Monaco, Monaco; Geovolco Team, Department of Earth Sciences, University of Geneva, Genève, Switzerland

## Abstract

Volcanic ash is a significant source of micronutrients including iron (Fe), copper (Cu), and zinc (Zn) in oligotrophic tropical waters. These bioactive metals enhance primary productivity, influencing local and global biogeochemical cycles. This study explores how volcanic ash exposure affects trace metal uptake and photophysiological response, and how redox-sensitive metal stable isotope measurements in the tissues of the scleractinian coral *Stylophora pistillata* can provide crucial information on coral health. Controlled coral culture experiments were conducted in which coral nubbins were exposed to varying intensity and duration of volcanic ash. Throughout the experiment, coral symbionts showed enhanced photosynthetic performance irrespective of intensity or duration of ash exposure. Stable isotopes, such as δ^65^Cu and δ^56^Fe, in the coral tissue are marked by systematic variations, not associated with intensity or duration of ash exposure. Instead, we suggest biologically modulated redox-sensitive fractionation associated with ash exposure, linked to the coral host’s oxidative stress state. This is evidenced by significant correlations between δ^65^Cu in coral hosts and photophysiology, with lighter Cu isotope ratios associated with higher photosynthetic performances. Hence, we propose that δ^65^Cu, and more generally redox-sensitive isotopic ratios (i.e. δ^56^Fe), in coral hosts serves as an indicator of the physiological state of symbiotic corals.

## Graphical Abstract

**Graphical Abstract:**
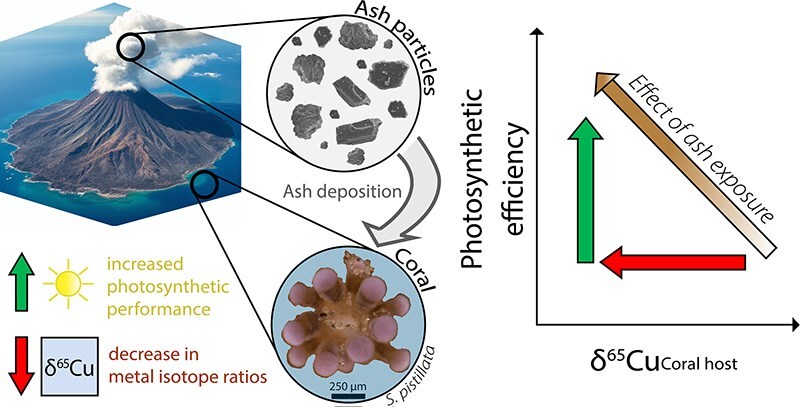

## Introduction

Atmospheric particles (aerosols) such as desert dust and volcanic ash play a crucial role in the global biogeochemical cycling of trace metals, with millions of tons of these aerosols transported and deposited annually across land and ocean surfaces [1]. Upon contact with seawater, these aerosols release essential trace metals, making them a significant source of nutrients in oligotrophic marine environments [2–6]. The leaching potential of aerosols (sum of individual metal solubilities) is affected by its mineralogical properties [7]. Next to desert dust, arguably a major source of aerosols is of volcanogenic origin. Explosive volcanic eruptions, although irregular and less frequent, are capable of emitting tons of tephra (fragmented magma in form of fine particles and rocks) into the atmosphere during an eruptive period [8]. Volcanic ash, the finest sized tephra with a particle size <2 mm, is able to be transported across thousands of kilometres before settling [9].

Volcanic ash leaches a wide range of major and minor elements into seawater [9], serving as a ‘micronutritional cocktail’ for marine biota, with bioactive metals such as iron (Fe), copper (Cu), and zinc (Zn) rapidly released from the ash particle surface into ocean surface waters [10–13]. Although the amount of leached elements is sample dependent, freshly erupted volcanic ash commonly supplies higher concentrations of water-extractable Fe and Cu (>5 mg/kg ash) [14], compared to Zn (<5 mg/kg ash) [9], but the leaching potential is largely governed by the chemical composition of the source magma, the ash particle size, and the surface salt coatings [2, 13, 15]. This leaching of metals from volcanic ash plays a vital role in marine nutrient dynamics, evidenced by the presence of unusually large phytoplankton blooms that follow volcanic eruptions [16–18] as a result of the alleviation of Fe and Mn limitation in ocean waters [18, 19].

Due to the wide dispersion range of ash fallout and the proximity of many active volcanoes to tropical reefs, many coral reefs will experience the effects of ash deposition in their lifetime (as projected for coral reefs in the coral triangle [20]). Recently, laboratory studies have demonstrated that volcanic ash exposure enhances coral physiology by stimulating the primary productivity of symbiotic living dinoflagellates of the family Symbiodiniaceae [21] within the coral tissue [22]. Symbiodiniaceae are important for coral energetics as they can provide up to 95% of the coral’s daily energy requirements [23]. These symbionts are known to actively take up trace metals such as Fe, Cu, or Zn [24, 25], and even minor increases in these trace metals can directly enhance the symbiont’s photosynthesis, increase coral resilience, and boost coral health [26–28].

Fe, Cu, and Zn are essential for coral physiology, as they act as metal co-factors in key proteins and enzymes, particularly in photosynthesis and antioxidant systems for both the coral host and its symbiotic algae [29]. Fe is especially important in coral photosynthesis, involved in chlorophyll synthesis [30] and in the electron transport chain [31]. It also plays a role in antioxidant enzymes such as the superoxide dismutase (SOD), with its uptake depending on Cu and Zn availability [32]. Cu, in multiple oxidation states, supports electron transfer in photosynthesis and respiration [33, 34], while Zn enhances enzymatic activity [35] and has structural roles [36], such as in carbonic anhydrase, important for coral skeleton formation [37]. Maintaining optimal levels of trace metals, such as Zn and Cu, is crucial for coral health due to their dual roles as essential nutrients and potential toxins. However, balancing metal levels is critical, as both deficiencies and excesses can disrupt symbiotic partnerships and metabolic processes [38]. Symbiotic corals may redistribute metals to compensate for deficiencies [32, 38], yet there are limits. Elevated metal levels can enhance the production of harmful radical oxygen species (ROS) [39, 40], leading to oxidative stress when antioxidant capacities are overwhelmed.

The constant production of ROS as a by-product of electron transport in chloroplasts [41] necessitates robust antioxidant defences, to scavenge ROS, which is critical in managing oxidative stress. Cu/Zn SOD, with its high efficiency and fast turnover rate [42], is a key component in symbiotic corals, guiding superoxide radicals to the Cu reaction centre and highlighting Cu’s crucial role in the coral antioxidant defence system [43, 44]. The antioxidant enzyme is located in the chloroplast stroma and converts superoxide radicals (O_2_^.−^) into molecular oxygen (O_2_) and hydrogen peroxide (H_2_O_2_), thereby reducing the risk of cellular damage from reactive oxygen species. However, when metal loading surpasses the coral’s capacity to manage oxidative stress, ROS accumulation can cause lipid peroxidation, protein oxidation, and DNA damage, ultimately triggering coral bleaching through the expulsion of symbionts and further compromising coral health [45, 46].

The Cu, Zn, and Fe isotopic composition (δ^65^Cu, δ^66 ^Zn, and δ^56^Fe) recently proved highly powerful for disease’s diagnosis and prognosis in humans [47–50] including cancers [51] and neurodegenerative disorders [52–54]. They also proved to be reliable markers of the metabolic health status and normal aging conditions [55, 56] as shown in mammals (i.e. mice) and in *Caenorhabditis elegans* worms and, more recently, proved to be highly promising to assess adverse health effect induced by chronic volcanic ash exposure in mice [57].

For corals, metal isotope data are scarce, although they proved to be highly promising. A pioneering study by Ferrier-Pagès *et al*. [25] highlighted the effect of thermal stress and heterotrophic feeding on the isotopic fractionation of δ^65^Cu and δ^66^Zn in symbionts and in the host tissue of *Stylophora pistillata*. Building on these results, there have been advances in the field, as Cu and Zn isotopes in shallow-water coral skeletons have been analysed [58, 59]. Nevertheless, stable metal isotope analysis on coral soft tissue is rare, and while the effects of Fe, Cu, and Zn enrichments on coral physiology are well-documented [e.g. 26, 27], understanding of the mechanisms behind stable isotope fractionation of these metals still remains limited [60]. So far, only few studies investigated the mechanistic pathways accounting for metal stable isotopic fractionation in corals, although it is well-established that unaccounted for biological processes significantly influence isotope fractionation, as seen in various calcareous tissues of marine organisms [e.g. 58, 61].

In this study, we investigate the effects of varying volcanic ash quantities and exposure duration on nubbins of *S. pistillata*, which have been previously shown to enhance symbiont photochemical reactions when exposed to volcanic ash [22]. We specifically examined Fe, Cu, and Zn—essential metals known to significantly impact symbiont photosynthesis—to determine whether and how ash exposure and duration alter metal uptake in coral host and its symbionts, as well as their stable isotope compositions. By linking metal isotope fractionation to symbiont photosynthetic efficiency under ash exposure, we aim to determine the extent to which these processes are biologically driven. These findings will provide insights in the applicability of metal isotope ratios in coral research and enhance our understanding of how aerosol micronutrient supply impacts the coral metallome and coral resilience.

## Materials and methods

### Volcanic ash

Freshly erupted volcanic ash, originating from the Vulcanian eruption of the La Soufrière volcano on St. Vincent, was collected on 9 April 2021 from a balcony in central Barbados by Dr John B. Mwansa (Airy Hill, Barbados). The ash was gathered in plastic trays on the day the eruption occurred, and was stored in zip bags, following protocols outlined by Witham *et al*. [62] and Stewart *et al*. [63]. The volcanic ash of andesitic–basaltic composition consists primarily of silicate glass and plagioclase feldspar, an aluminium feldspar mineral. Leaching experiments reveal low concentrations of potentially toxic elements in the water-soluble fraction [64]. In this study, pristine volcanic ash and ash leached for 24 h in deionized water in a ratio of 0.3 g in 30 ml were analysed for their trace metal composition.

### Coral culture experiments

**Figure 1.**
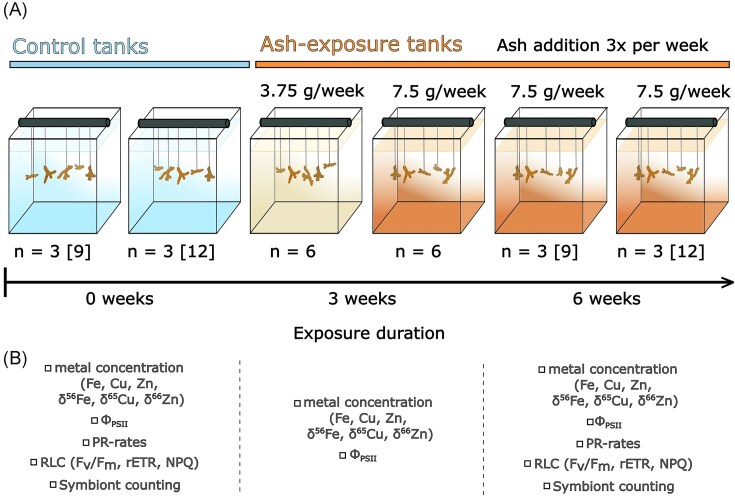
(A) Experimental setup. The colour gradient in the tanks indicates the intensity of ash exposure. Nubbins of *Stylophora pistillata* were maintained under constant light intensity and water temperature. Control tank nubbins were sampled simultaneously with the 7.5 g/week condition at the end of the 6-week period. *n* represents the number of analysed nubbins (*n* = 6 per condition), with the number in brackets [] showing the total number of nubbins in the tank. The lower part (B) lists the measurements performed on nubbins from the corresponding tanks.

A detailed description of the coral culture conditions is given in Förster *et al*. [22]. Briefly, 54 coral fragments (‘nubbins’, ∼2–4 cm in size) were cut prior to the experiment with a bone clamp from large parent colonies of the branching coral *S. pistillata*, which were consistently reared in culture conditions (light: 200 ± 10 µmol photons/m^2^/s; temperature: 25°C ± 0.2°C; pH: 7.93 ± 0.04; salinity: 38.5) at the Centre Scientifique de Monaco (Monaco). The scleractinian coral *S. pistillata* has been widely used as a model species [65] and was chosen due to its well-known responses to various metals [26–28, 66–69]. After a 4-week healing period in which nubbins were fed twice a week with *Artemia salina* nauplii, feeding was stopped and nubbins were distributed across six (30 l) tanks and subjected to four different exposure conditions: one non-exposed control (21 nubbins in two tanks) and three ash treatments with varying amounts and duration of volcanic ash (Fig. 1). In the first two ash treatments (six nubbins per condition, one tank per treatment), nubbins were exposed to 3.75 or 7.5 g of ash per week for 3 weeks (ash treatments 1 and 2, respectively). In the third ash treatment (21 nubbins in two tanks), they were exposed to 7.5 g of ash per week for 6 weeks. Both the control and the third ash exposure condition were replicated to account for potential tank effects, which were considered negligible due to statistically indifferent Φ_PSII_ results (Table S2).

While the maximum exposure duration resembles the median eruption time of 7 weeks [70], eruption timelines may vary significantly. For instance, the 2021 eruption of La Soufrière in St. Vincent had a 13-day explosive phase [71], while the 1997 eruption of Soufriere Hills in Montserrat featured two explosive phases lasting 8 and 30 days, with intervals between explosions ranging from 2.5 to 63 h [72]. Three times a week, unfiltered volcanic ash (ash treatment 1: 1.25 g; ash treatment 2 and 3: 2.5 g) was added into the experimental tanks, totalling 3.75 g/week for ash treatment 1 and 7.5 g/week for ash treatments 2 and 3. The amount of ash in the experimental conditions was selected based on its limited effect on shading and to prevent coral mortality. Each morning, before ash addition, the seawater inlet to the tanks was turned off. After 8 h, the seawater supply was restored for complete water renewal overnight. Nubbins in ash treatments 1 and 2 were frozen at −20°C after 3 weeks, while control nubbins and those in ash treatment 3 were stored at −20°C after 6 weeks for further analysis.

### Photophysiological analyses

#### Chlorophyll fluorescence measurements

Chlorophyll *a* fluorescence measurements allow us to quantify the efficiency of photoreactions occurring in photosystem II (PSII) [73], which plays a crucial role in the oxygenic photosynthesis. PSII is a protein–pigment complex, located in the thylakoid membranes of chloroplasts, that catalyses the light-driven electron transfer by splitting water into protons, electrons, and molecular oxygen [74]. Chlorophyll *a* fluorescence of PSII was measured twice weekly at a consistent time throughout the experiment using a Pulse-Amplitude-Modulated (PAM) fluorometer (DIVING-PAM, Walz®, Germany) on six nubbins per condition. Minimal (F_0_) and maximal (F_m_) fluorescence yields at 200 µmol photons m^2^/s (ambient light setting) were recorded using a weak pulsed red light (max. intensity <1000 µmol photons/m^2^/s, 3 ms width, 0.6 kHz frequency) and a saturating pulse of actinic light (max. intensity >8000 mmol photons/m^2^/s, 800 ms width), respectively. The effective quantum yield (Φ_PSII_), which monitors the real-time activity of PSII, reflects the coral’s photophysiological state and the energy used in photochemistry [75].

At the end of the 6-week experiment, rapid light curves (RLCs) were generated from dark-adapted nubbins (six from control and ash treatment 3) by illuminating the corals with 10-s light pulses at 11 increasing intensities, ranging from 0 to 1956 µmol photons/m^2^/s. The maximum photosynthetic efficiency in dark-adapted corals (F_v_/F_m_), non-photochemical quenching (NPQ), and the relative electron transport rate (rETR) were used to evaluate PSII efficiency across treatments [75]. During measurements, the fibre-optic probe was positioned perpendicular to the coral surface at a fixed distance of 5 mm. The recorded Φ_PSII_, F_v_/F_m_, rETR, and NPQ were plotted as a function of PAR and visualized by using the WinControl_v.3.29 program (Walz GmbH®, Effeltrich, Germany).

#### Rates of photosynthesis and respiration

At the end of the experiments, rates of net photosynthesis (P_n_) and respiration (R) were determined for six nubbins from both the control and ash treatment 3 (*n* = 3 per tank). Each nubbin was placed in 60 ml Plexiglass® chambers containing 0.45-µm-filtered seawater, maintained at 25°C and continuously stirred using magnetic stirrers. P_n_ was measured under 200 µmol photons/m^2^/s irradiance, while R was measured in the dark, both for a duration of roughly 45 min. Oxygen concentration was continuously monitored using a polymer optical fibre sensor (PreSens®) connected to an Oxy-4 fibre-optic oxygen meter (PreSens®) and recorded with the Oxy4v2-30fb software. Before measurements, sensors were calibrated using seawater saturated with 100% O_2_ and 0% O_2_ (N_2_-saturated) at ambient air pressure.

Following the measurement, the nubbins were stored at −20°C for the symbiont quantification. Frozen nubbins were stripped of soft tissue using a jet of compressed air and 0.22-µm-filtered seawater from an air brush. The resulting extract containing both host tissue and symbionts was homogenized for 20 s using an Ultra-Turrax T25 [Janke & Kunkel (IKA)®]. The homogenate was then divided into three 100 µl subsamples, each diluted in 9.9 ml Isoton II (BeckmanCoulter®, Ic, 8546719). Symbiont counts were obtained using a Z1 Coulter Particle Counter (Beckman Coulter®), while only particles larger than 7 µm were identified and counted as symbionts.

P_n_ and R rates were normalized to both the coral surface area and the total symbiont count, expressed as µmol O_2_/h/cm^2^, or pmol O_2_/h/symbiont cell, respectively. Coral surface area was determined on the cleared skeletons using the single-dip wax technique [76].

### Trace metal analysis

#### Iron, copper, and zinc concentrations in coral soft tissue and volcanic ash

Elemental concentrations were measured in the coral host, its symbionts, pristine volcanic ash, and in ash leached in de-ionized water for 24 h. Three nubbins from each tank (a total of three from ash treatments 1 and 2, and six from the control and ash treatment 3) were collected after either 3 or 6 weeks and stored at −20°C. Coral tissue and symbionts were separated from the skeleton using an airbrush with pressurized air and deionized water. The mixture was homogenized for 20 s using an Ultra-Turrax T25 [Janke & Kunkel (IKA)®] and centrifuged three times at 8000 *g* for 10 min at 4°C. After each centrifugation, the supernatant containing the coral tissue was transferred to a separate 50 ml conical tube, while the symbiont containing pellet was resuspended in deionized water. Both the coral host tissue and symbionts were then freeze-dried for subsequent major and trace element analysis.

Major and trace element analyses were conducted at the Laboratoire Magmas et Volcans (LMV) in clean laminar flow hoods using double-distilled acids to avoid exogeneous contamination. The first step consisted of dissolving the samples in a mixture of concentrated acids. In brief, coral (host tissue and symbionts) and ash samples were dissolved in a concentrated HNO_3_–H_2_O_2_ and HF–HNO_3_–HCl mixture, respectively, in Savillex beakers, first at room temperature and then at 100°C for at least 72 h. To guarantee complete dissolution, this process was repeated three times. Solutions were then evaporated at 80°C and diluted in 0.5 N HNO_3_ to obtain a ‘mother’ solution. Only for major elemental analyses in ash samples, a different digestion protocol was used. Samples were melted with lithium metaborate and then, metaborate fusion products were dissolved in nitric acid (0.5 N). In parallel, loss on ignition was measured by weighing the bulk sample before and after 1 h of calcination at 1000°C. After complete dissolution, major and trace element concentrations were measured on QQQ-ICP-MS (Agilent 8900) and ICP-OES (Agilent 5800), respectively, following a similar theoretical procedure to that described in Sauzéat *et al*. [57]. Briefly, two weighed aliquots of the ‘mother’ solution were diluted with 2% v/v HNO_3_ to obtain a dilution factor of about 2500 and 5000 for major and trace elements, respectively—a value chosen to minimize matrix effects while maintaining sufficient volume and signal for all measured elements and leaving enough solution available for subsequent isotopic ratio measurements. Calibration of the signal was carried out using blanks and various dilutions of multi-element standard solutions and, if necessary, instrumental drift was corrected using indium (In) as an internal standard for trace elemental measurements. For trace analyses, highly interfered elements (m <75) were acquired using a helium or oxygen flux through the collision/reaction cell, while non-interfered elements (m >75) were measured in no-gas mode. Accuracy and precision were assessed with matrix-matched certified reference materials (biological Standard Reference Material (SRM): NIST 1577c; geological SRM: BHVO-1, BHVO-2, DR-N), re-run analyses, and complete duplicate sample measurements. The accuracy for Fe [85.4% ± 8.3% (SD)], Cu [88.4% ± 7.9% (SD)], and Zn [87.9% ± 7.6% (SD)] relative to the GeoRem preferred values for NIST 1577c, and for Fe [101.9% ± 0.4% (SD)], Cu [101.9% ± 4.8% (SD)], and Zn [107.8% ± 5.7% (SD)] relative to the certified values of BHVO-1, BHVO-2, and DR-N, aligned with the uncertainty of previously published results, demonstrating reproducibility on average ∼10% variation. Metal concentrations in coral host and symbionts are reported in µg/g dry weight.

#### Iron, copper, and zinc isotopic composition

Fe, Cu, and Zn isotopic compositions were analysed in subfractions of freeze-dried coral host, their symbionts (*n* = 3 per tank), and in pristine and leached volcanic ash. The determination of isotopic compositions was carried out according to Maréchal *et al*. [77] for Cu and Zn and to Poitrasson and Freydier [78] for Fe at the facilities of the LMV. The fully detailed analytical protocol can be found in Sauzéat *et al*. [79] and Sauzéat *et al*. [57]. Briefly, after digestion, the samples were evaporated to dryness and the residue was redissolved in a mixture of HCl (8 M) + H_2_O_2_ (0.001%). Cu, Fe, and Zn were then isolated and purified by ion-exchange chromatography using 1 ml of AG-MP1 anion exchange resin following a revised procedure from Costas-Rodríguez *et al*. [80] and fully described by Sauzéat *et al*. [79]. Copper, zinc, and iron isotopic compositions were then measured on a Thermo Scientific Neptune Plus^TM^ MC-ICP-MS instrument (LMV, France) in wet plasma conditions at concentrations of ∼250 and 500 µg/l, respectively, either in medium mass resolution for Cu and Zn isotope ratios or high mass resolution for Fe. The Cu, Zn, and Fe isotope ratios are expressed in delta notation (δ^65^Cu, δ^66^Zn, and δ^56^Fe, per mil, ‰) relative to standards NIST SRM 976 for Cu, JMC 3-0749 L for Zn and IRMM14 as follow:


\begin{eqnarray*}
\delta {}_{\mathrm{\,\,}}^{{\mathrm{XX}}}{A_{\textit{sample}}} = \left[ {\frac{{{{\left( {{\raise0.7ex\hbox{${{}_{\mathrm{\,\,}}^{{\mathrm{xx}}}A}$} \!\mathord{\left/ {\vphantom {{{}_{\mathrm{\,\,}}^{{\mathrm{xx}}}A} {{}_{\mathrm{\,\,}}^{{\mathrm{xy}}}A}}}\right.} \!\lower0.7ex\hbox{${{}_{\mathrm{\,\,}}^{{\mathrm{xy}}}A}$}}} \right)}_{\textit{sample}}}}}{{{{\left( {{\raise0.7ex\hbox{${{}_{\mathrm{\,\,}}^{{\mathrm{xx}}}A}$} \!\mathord{\left/ {\vphantom {{{}_{\mathrm{\,\,}}^{{\mathrm{xx}}}A} {{}_{\mathrm{\,\,}}^{{\mathrm{xy}}}A}}}\right.} \!\lower0.7ex\hbox{${{}_{\mathrm{\,\,}}^{{\mathrm{xy}}}A}$}}} \right)}_{std}}}} - 1} \right]{\mathrm{*}}{10^3}
\end{eqnarray*}

where ^XX^A and ^XY^A refer to ^65^Cu, ^66^ Zn, or ^56^Fe and ^63^Cu, ^64^ Zn, or ^54^Fe, respectively.

For Cu and Zn isotope ratios, instrumental mass bias correction relied on a combination of external correction in a sample-standard bracketing approach (SSB) and internal mass fractionation (exponential law) using Zn and Cu elemental doping, while a single SSB correction method was applied for Fe isotope ratios. The overall procedure led to total procedural blanks (*n* = 2) of 0.8 ng for Cu, 9.1 ng for Zn, and 15.1 ng for Fe, which represents <0.8% of the amount of each element present in the measurement solutions. The long-term precision of the results was assessed by sample re-run analysis and repeated measurements of the bracketing standards run every two samples for Cu and Zn and every sample for Fe. Sample accuracy was assessed by the measurement of certified biological and geological reference materials (1577c, BHVO-2). The long-term external standard reproducibility (2 SD) was 0.1‰ (*n* = 40) for Cu and Zn, and of 0.17‰ (*n* = 80) for Fe (Table S1), and our results of the certified reference materials (1577c and BHVO2) are in good agreement with certified values for all isotope ratio measurements [on average ±0.11‰ (2 SD, *n* = 10) for δ^65^Cu, ±0.12‰ (2 SD, *n* = 10) for δ^56^Fe, and ±0.03‰ (2 SD, *n* = 10) for δ^66^Zn]. Given our long-term precision and the accuracy obtained on reference material measurements, the 2 SD analytical uncertainty adopted in this study is of 0.1‰ for δ^65^Cu and δ^66^Zn and of 0.15‰ for δ^56^Fe.

### Statistical analysis

Statistical tests were performed using RStudio Version 4.2.0 [81]. The Shapiro–Wilk test assessed the normality of photophysiological data (F_v_/F_m_, rETR, NPQ, photosynthesis, and respiration rates) while homoscedasticity was verified using Levene’s test. Depending on the outcome, a single one-way Analysis of Variance (ANOVA) was applied for normally distributed data, or the Wilcoxon–Mann–Whitney test (WMW) was performed for non-normal data. If the homoscedasticity test was rejected but normality was met, the Welch *t*-test (Welch) was applied. Due to limited sample sizes in trace metal data for coral tissue (*n* = 6 for control and ash treatment 3; *n* = 3 for ash treatments 1 and 2), the non-parametric Kruskal–Wallis test (KW) was performed to analyse the Fe, Cu, and Zn concentrations, as well as δ^56^Fe, δ^65^Cu, and δ^66^Zn isotopic ratios. Dunn’s post hoc test, with Holm–Bonferroni correction for *P*-value adjustment [82], was employed to identify significant differences across the four experimental conditions. Statistical significance was set at *P*-value < .05, with significance levels denoted as *P*-value > .05 (not significant), *P*-value ≤ .05 (*), *P*-value ≤ .01 (**), and *P*-value ≤ .001 (***).

## Results

### Photophysiology

#### Effective quantum yield of PSII

**Figure 2.**
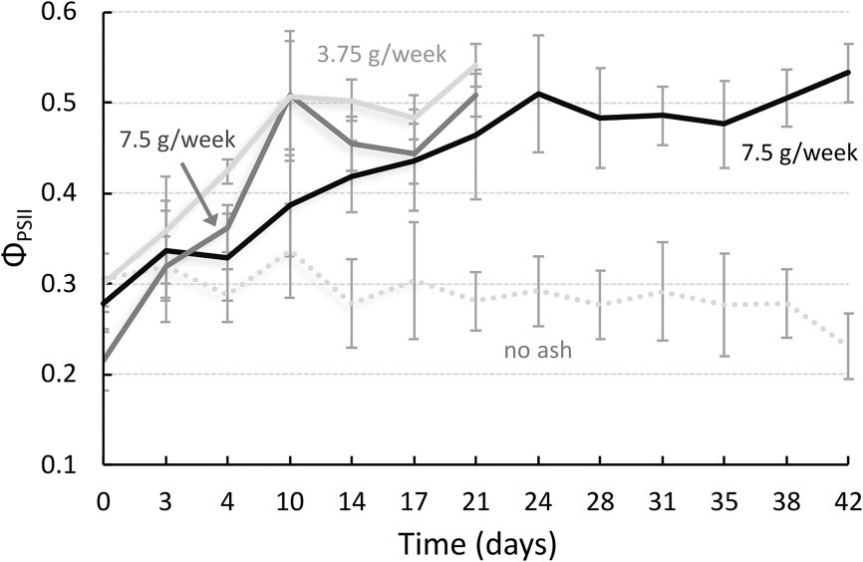
Effective quantum yield of PSII (Φ_PSII_) of *Stylophora pistillata* maintained under four different exposure conditions: no ash (*grey dotted*), 3.75 g/week for 3 weeks (*light grey*), 7.5 g/week for 3 (*dark grey*) and 6 weeks (*black*) (*n* = 6 per condition). Data are presented as mean ± SD.

Measurements of the effective quantum yield of PSII (Φ_PSII_), which was measured under a light level of 200 µmol photons/m^2^/s, revealed a similar value for all experimental conditions at the start of the experiment (Φ_PSII _≈ 0.3; Fig. 2). While control nubbins maintained the initial Φ_PSII_ throughout the experiment, the monitored Φ_PSII_ over 42 days revealed a similar chlorophyll fluorescence change in all three volcanic ash treatments. The addition of 3.75 and 7.5 g ash per week led to a steady increase of Φ_PSII_ over the entire experiment, approaching a Φ_PSII_ value of 0.54 ± 0.01 and 0.51 ± 0.02, respectively, after 3 weeks. Additionally, the consistency of results across tanks of the same condition demonstrated that minor variations in tank conditions, such as temperature and irradiance, did not significantly affect Φ_PSII_ measurements (e.g. two tanks in non-exposed conditions, KW, post hoc, *P* = .7; Table S2).

#### Photophysiological response of nubbins exposed to 7.5 g/week for 6 weeks

The results of the RLC-derived photochemical parameters (F_v_/F_m_, rETR, and NPQ), along with photosynthesis and respiration rates in control and corals exposed for 6 weeks to 7.5 g/week, have been previously published [22]. Here, we present these findings again in the context of the observations in chapter 3.3.

After 6 weeks, RLCs performed on dark-adapted nubbins exposed to 7.5 g volcanic ash per week revealed 58%–113% higher F_v_/F_m_ at low irradiance (0–220 µmol photons/m^2^/s) compared to control nubbins (Table S3). At high light intensity (>664 µmol photons/m^2^/s) control nubbins showed 6%–21% higher F_v_/F_m_ values than ash-exposed nubbins (Photosynthetically Active Radiation (PAR) = 1032, ANOVA, *P* < .042; PAR = 1956, ANOVA, *P* < .016; Table S3). A similar irradiance-depending trend was observed in the ETR for PSII. Ash-exposed nubbins demonstrated significantly (67%–83%) higher rETR at lower irradiances (10–220 µmol photons/m^2^/s). At high irradiance levels, they appeared to pump fewer electrons through the electron transport chain compared to control nubbins (PAR = 1032, ANOVA, *P* < .039; PAR = 1956, ANOVA, *P* < .015; Table S4).

NPQ, which quantifies the conversion of excess energy into heat, was indistinguishable for both ash-exposed and control nubbins at low irradiance levels (0 and 10 µmol photons/m^2^/s). However, as light intensity increases, NPQ levels in control nubbins consistently surpassed those in ash-exposed nubbins, with the greatest difference observed at PAR = 1956 (ANOVA, *P* < .00002; Table S5).

Ash-exposed nubbins increased their net oxygen production (P_n_) to 0.44 µmol O_2_/h/cm^2 ^± 0.08 (SD) during the incubation, while control nubbins produced 0.17 µmol O_2_/h/cm^2^ ± 0.07 (SD) (ANOVA, *P* < .0001; Table S6). On average, symbionts experiencing a constant ash supply for 6 weeks produced 0.21 pmol O_2_/h ± 0.04 (SD), nearly double the amount produced in contrast to symbionts not exposed to ash [0.11 pmol O_2_/h ± 0.05 (SD), ANOVA, *P* < .004; Table S6]. Respiration was statistically identical in control [0.42 µmol O_2_/h/cm^2 ^± 0.13 (SD)] and ash-exposed conditions [0.32 µmol O_2_/h/cm^2 ^± 0.04 (SD)] when normalized to skeletal surface area (ANOVA, *P* = .1; Table S6). However, when normalized to symbiont cells, control nubbins revealed a 70% higher respiration [0.27 pmol O_2_/h/symbiont cell ± 0.24 (SD)] compared to ash-supplied corals (0.16 pmol O_2_/h/symbiont cell ± 0.02 (SD), Welch, *P* < .02; Table S6].

### Trace element concentration and isotopic composition

#### Pristine and leached volcanic ash

The volcanic ash from the La Soufrière eruption on St. Vincent was analysed for its major and trace element concentrations including Fe, Cu, and Zn content, along with their stable isotopic ratios (Table S7). The entire chemical characterization of major and trace elements of the pristine and leached volcanic ash is listed in Table S7. The bulk composition of both pristine (unaltered) and leached volcanic ash exhibits similar metal concentrations. For example, Fe, measured as Fe_2_O_3_, is present at 7.5 wt.% ± 0.32 (2 SD, *n* = 2) in pristine ash and 7.2 wt.% ± 0.04 (2 SD, *n* = 2) in leached ash. Zn concentrations measured in pristine ash are 248.41 µg/g ± 53.40 (2 SD, *n* = 2) equivalent to the Zn content in leached ash of 304.28 µg/g ± 39.54 (2 SD, *n* = 2). Cu levels are 116.35 µg/g ± 25.54 (2 SD, *n* = 2) in the pristine and 128.91 µg/g ± 13.50 (2 SD, *n* = 2) in the leached ash. δ^56^Fe, δ^65^Cu, and δ^66^Zn—stable metal isotope ratios are similar between bulk pristine and leached volcanic ash. δ^56^Fe in pristine ash is −0.21‰ ± 0.04‰ (*SD*) and −0.22‰ (SD <0.01) in leached ash. δ^65^Cu is consistent in both ash types at +0.24‰ ± 0.04‰ (SD, *n* = 2). δ^66^Zn measures +0.31‰ (*n* = 1) in pristine ash and +0.34‰ (*n* = 1) in leached ash.

#### Coral metallome

The total Fe, Cu, and Zn concentrations and the δ^56^Fe, δ^65^Cu, and δ^66^Zn isotope ratios for control corals and nubbins exposed to three different volcanic ash conditions are listed in Table 1. Symbionts generally increased their mean metal concentrations by 26%–170% compared to the coral host, with metal content varying both within and between conditions, although the difference between metal loads of symbiont and host was not significant (Table S8). Metal concentrations in the host tissue and symbionts between control and ash-exposed conditions were not significantly different, although trace metal overloads were still observed after ash exposure. For example, Fe concentration increased significantly by 178% after 3 weeks of 3.75 g/week [120.58 µg/g ± 11.29 (SD)], compared to the control tissue [43.39 µg/g ± 8.84 (SD); KW, post hoc, *P* < .005; Table S9). Similarly, symbionts tended to accumulate Fe and to a lesser extent Zn, as shown in the 3-week, 7.5 g/week exposure condition (Fe: KW, post hoc, *P* < .016; Table S9). Zn concentrations in symbionts also showed a significant decrease of 68% from the 3-week, 7.5 g/week to the 6-week, 7.5 g/week ash exposure conditions (KW, post hoc, *P* < .032; Table S9). Cu concentrations in both tissue and symbionts remained statistically unaffected by varying volcanic ash loadings.

**Table 1.** Micronutrient concentrations of Fe, Cu, and Zn (µg/g dry weight) and their isotope ratio (‰) in host tissue and in symbionts of *Stylophora pistillata* maintained under four different volcanic ash exposure conditions.

Ash exposure	Sample	Fe (µg/g)	Cu (µg/g)	Zn (µg/g)	δ^56^Fe (‰)	δ^65^Cu (‰)	δ^66^Zn (‰)
None (*n* = 6)	Coral host	43.29	11.82	128.82	−0.49	0.72	0.31
	*SD*	*8.84*	*1.30*	*21.83*	*0.19*	*0.05*	*0.02*
	Symbionts	69.27	17.27	248.04	−0.61	0.54	0.41
	*SD*	*47.36*	*12.79*	*200.52*	*0.19*	*0.15*	*0.04*
3.75 g/week 3 weeks (*n* = 3)	Coral host	120.58	10.18	155.90	−0.69	0.59	0.33
	*SD*	*11.29*	*1.55*	*29.51*	*0.04*	*0.01*	*0.01*
	Symbionts	146.37	9.04	292.65	−0.40	0.53	0.36
	*SD*	*30.97*	*2.88*	*24.15*	*0.12*	*0.10*	*0.04*
7.5 g/week 3 weeks (*n* = 3)	Coral host	65.72	8.88	127.62	−0.68	0.60	0.35
	*SD*	*29.89*	*3.73*	*68.29*	*0.02*	*0.08*	*0.03*
	Symbionts	279.61	17.96	406.38	−0.34	0.48	0.42
	*SD*	*84.09*	*5.32*	*113.12*	*0.02*	*0.07*	*0.02*
7.5 g/week 6 weeks (*n* = 6)	Coral host	71.47	14.50	155.63	−0.41	0.63	0.34
	*SD*	*24.39*	*7.01*	*72.53*	*0.03*	*0.03*	*0.04*
	Symbionts	98.19	9.56	130.15	−0.46	0.48	0.43
	*SD*	*51.88*	*5.59*	*63.00*	*0.21*	*0.09*	*0.04*

**Figure 3.**
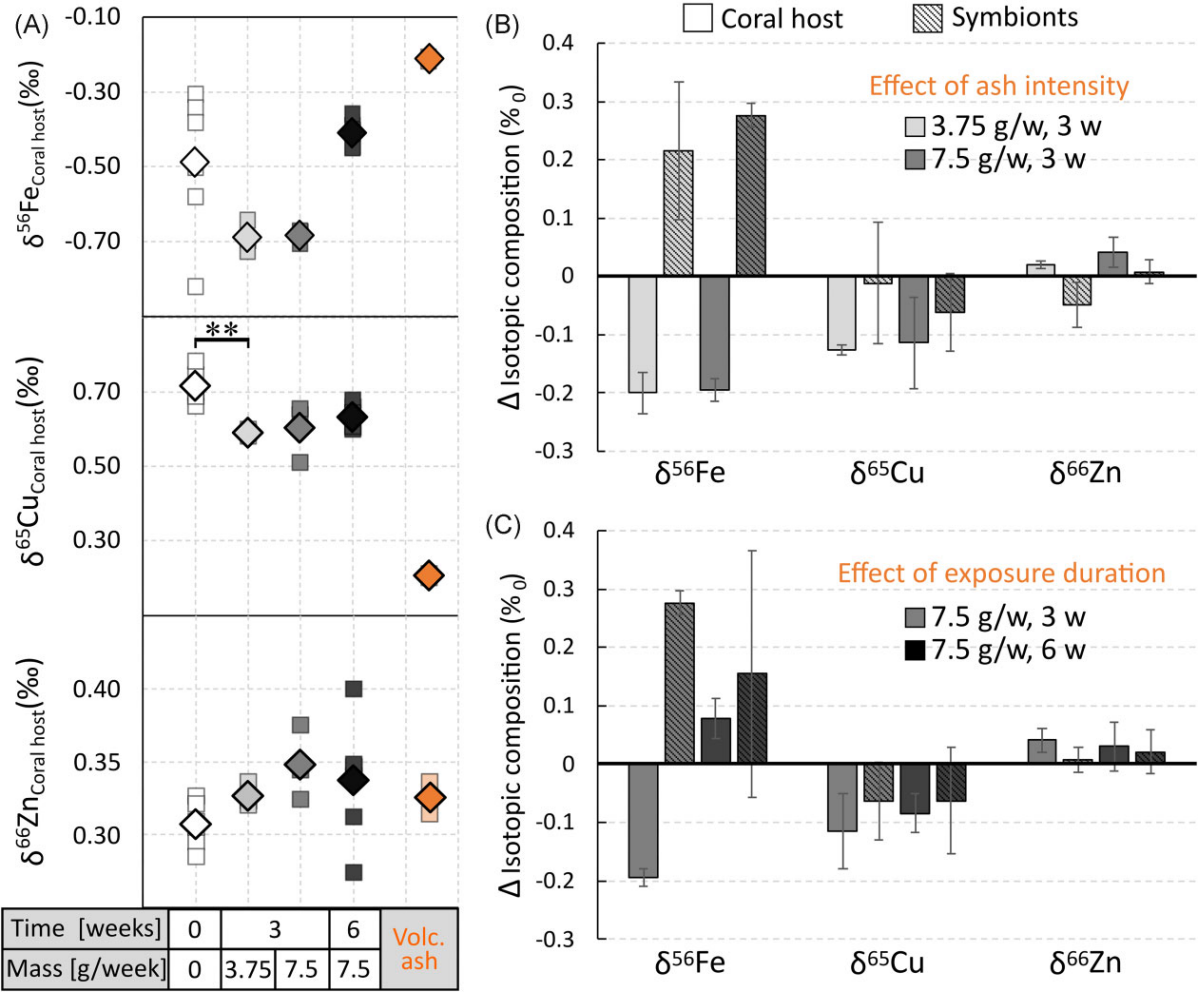
Effects of ash intensity on metal isotope ratios in the soft tissue of *Stylophora pistillata*. (A) δ^56^Fe, δ^65^Cu, and δ^66^Zn in the host tissue maintained under four ash exposure conditions. Data are presented as the mean (◇) of individual measurements in host tissue (□). Effects of (B) ash loading and (C) exposure duration on δ^56^Fe, δ^65^Cu, and δ^66^Zn in symbionts and coral host. Data are presented as the deviation of the means of ash-exposed conditions relative to the non-exposed (∆) ± SD. The white-to-black colour gradient resembles the intensity of ash exposure. Statistically significant differences between treatments are represented by asterisks (*), with an indication of the level of significance in the number of asterisks.

Generally, within a single condition, there was no difference in the isotopic composition of δ^56^Fe, δ^65^Cu, and δ^66^Zn between host tissue and symbionts (Table S8). Only δ^65^Cu was increased by 33% in the host tissue, compared to the symbionts in the non-exposed condition (KW, post hoc, *P* < .046), while, in contrast, δ^66^Zn was 34% higher in symbionts than in control tissue (KW, post hoc, *P* < .01; Table S9). Between different ash-exposed treatments, in symbionts, there was no systematic variation in δ^56^Fe, δ^65^Cu, and δ^66^Zn, except for δ^66^Zn (Fig. S1) exposed for 3 weeks to 3.75 g/week [0.36‰ ± 0.04‰ (SD)] and 6 weeks to 7.5 g/week [0.43‰ ± 0.04‰ (SD)] (KW, post hoc, *P* < .041; Table S9). Host tissues, however, were marked by more pronounced and systematic changes. This translated into an overall 10% increase in δ^66^Zn and a 12%–18% decrease in δ^65^Cu in coral host after ash exposure (Fig. 3A), as for example shown by significant differences between control tissue [δ^65^Cu = 0.72‰ ± 0.05‰ (SD) vs 0.59‰ ± 0.01‰ (SD) in the tissue after 3-week, 3.75 g/week; KW, post hoc, *P* < .007; Table S9]. Similarly, δ^56^Fe decreased by ∼40% in host tissues exposed over 3 weeks to volcanic ash, but then get back to non-exposed values after 6 weeks of exposure (Fig. 3A).

### Significant negative correlations of photosynthetic parameters with δ^65^Cu_Coral host_

**Figure 4.**
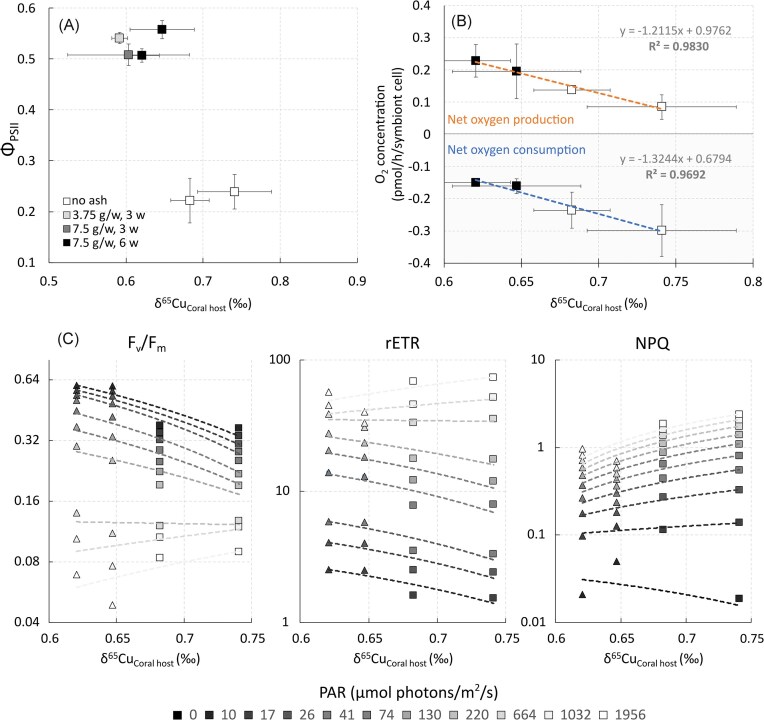
Relationship between δ^65^Cu_Coral host_ in *Stylophora pistillata* and various photosynthetic parameters. (A) Effective quantum yield value of PSII (Φ_PSII_), with each point representing the mean of Φ_PSII_ per tank ± SD. (B) Net photosynthesis and respiration rates of non-exposed (□) and 6-week ash-exposed nubbins (**■**). Change in oxygen concentration per hour is normalized by the number of symbiont cells. Each point represents the mean oxygen production/consumption per tank ± SD (*n* = 3). (C) RLC-derived photochemical parameters: total effective quantum yield of PSII (F_v_/F_m_), relative electron transport rate (rETR), and non-photochemical quenching (NPQ) in dark-adapted corals. Each point (Δ for nubbins exposed for 6 weeks to 7.5 g/week, and □ for non-exposed corals) represents the mean value of the respective parameter ± SD (*n* = 3 per tank).

Φ_PSII_ measurements in light-adapted nubbins (Fig. 2) and photochemical parameters derived from RLC of dark-adapted nubbins (Fig. 4C) showed strong negative correlations with the δ^65^Cu_Coral host_ isotopic signature (Fig. 4), but not with δ^56^Fe_Coral host_, or δ^66^Zn_Coral host_. Specifically, higher δ^65^Cu_Coral host_ ratios typically corresponded to lower Φ_PSII_ (*R*^2^ = 0.72, *P* < .033; Fig. 4A; Table S10). The RLC-derived photochemical parameters (F_v_/F_m_, rETR, and NPQ) negatively correlated with higher δ^65^Cu_Coral host_ at irradiances up to 664 µmol photons/m^2^/s (Fig. 4C). However, this negative correlation disappeared at high light intensities. NPQ levels positively correlated with δ^65^Cu_Coral host_ when PAR exceeds zero, with higher NPQ values linked to elevated δ^65^Cu (Fig. 4C).

Significance levels of the correlation between RLC-derived parameters and the δ^65^Cu_Coral host_ were assessed using two different sample sizes. First, the analysis was conducted on the means of three nubbins from a single tank, with two tanks for each condition (non-exposed and 6-week exposure to 7.5 g/week; *n* = 4). This approach yielded strong but non-significant correlations (F_v_/F_m_: *R*^2^ ≈ 0.79, *P* ≈ .11; rETR: *R*^2^ ≈ 0.78, *P* ≈ .11; and NPQ: *R*^2^ ≈ 0.83, *P* ≈ .08; Table S11). When correlating individual measurements on three nubbins per tank across four tanks (two tanks for each non-exposed and 6-week exposure to 7.5 g/week condition; *n* = 12) with δ^65^Cu_Coral host_, this analysis resulted in less strong but statistically significant correlations (F_v_/F_m_: *R*^2^ ≈ 0.52, *P* < .011; rETR: *R*^2^ ≈ 0.49, *P* < .011; and NPQ: *R*^2^ ≈ 0.45, *P* < .02;Table S11).

Furthermore, δ^65^Cu_Coral host_ exhibited a significant correlation with the net oxygen production (photosynthesis) and consumption rate (respiration) of the corals, normalized per symbiont cell (P_n_: *R*^2 ^= 0.98, *P* < .01; R: *R*^2^ = 0.97, *P* < .017; Fig. 4B; Table S10). Normalized per surface area, the net oxygen production correlated significantly with the δ^65^Cu_Coral host_, but not the consumption rate (P_n_: *R*^2^ = 0.98, *P* < .013; R: *R*^2 ^= 0.65, *P* = .2; Fig. S2a and Table S10). In contrast, photosynthesis and respiration rates did not correlate with δ^65^Cu_Symbionts_ (surface area: P_n_: *R*^2^ <<0.01, *P* = .98; R: *R*^2 ^= 0.24, *P* = .51; symbiont cell: P_n_: *R*^2^ << 0.01, *P* = 0.99; R: *R*^2^ << 0.01, *P* = 0.99; Fig. S2b and c and Table S10).

To understand the impact of data variability for each experimental condition, we bootstrapped the raw data with replacement to understand the variability in the mean values. For each experimental tank, we resampled the mean values for oxygen production rate normalized per symbiont cell and δ^65^Cu_Coral host_ and calculated the Pearson correlation coefficient (*r*). The resampling procedure was repeated 300 times, which provided a 90% credible range between −0.79 and −0.99 for *r*. The median value was −0.95, which is not as strong as the −0.99 observed in Fig. 4B. This is likely due to the limited number of observations per experimental condition, which have resulted in high standard deviations. Nevertheless, the bootstrapping approach reveals a strong and credible tendency toward high negative correlations (Fig. S3).

## Discussion

### δ^65^Cu_Coral host_ is linked to the photosynthetic efficiency of the endosymbionts

Once pristine volcanic ash mixes with seawater, the release of essential metals results in a rapid increase in primary production and growth of light-dependent organisms, such as the coral endosymbionts [22, 69]. The supply of essential trace metals overcomes enzyme-specific metal limitations, enhancing photochemical processes and leading to changes in F_v_/F_m_, rETR, NPQ, and photosynthetic rates after 6 weeks of exposure [this study, 22]. Biweekly measurements of Φ_PSII_ (Fig. 2) on ash-exposed nubbins across all experimental conditions reveal similar trends, indicating that the amount of ash does not play a crucial role in the photochemical efficiency of PSII of the symbionts. Even when the weekly ash amount was halved (7.5 g vs 3.75 g), the symbionts exhibited a similar photo response, indicating that certain metal thresholds can be overcome with even lower ash amount.

The exposure of *S. pistillata* nubbins to volcanic ash also resulted in a systematic shift to lighter redox-sensitive δ^65^Cu in the coral tissue (δ^65^Cu_Coral host_), as seen in Fig. 3A and Table 1. This isotopic shift closely aligns with the photophysiological state of the holobiont. The observed correlation between δ^65^Cu_Coral host_ and the coral physiological state could not be observed in the non-redox δ^66^Zn isotopic ratios in either the coral host or symbionts, highlighting a special role of Cu and more generally redox-sensitive metal isotope fractionation in coral health. The δ^65^Cu in the coral host is intrinsically linked to the photosynthetic activity of the endosymbionts (as seen in the ‘Significant negative correlations of photosynthetic parameters with δ^65^Cu_Coral host_’ section, Fig. 4). A low δ^65^Cu_Coral host_ value is associated with higher photosynthesis rates and enhanced photochemical reactions, such as increased electron transport through the electron transport chain and reduced energy dissipation as heat.

### Biologically induced isotope fractionation in *S. pistillata* rather than ash-derived isotopic signatures

Although metal leaching and biological uptake are evident, the metal content of nubbins exposed to volcanic ash does not systematically increase compared to control nubbins, although several signs of metal overloads are observed. For example, Fe content increases in both host and symbionts in response to ash exposure (coral host: no ash vs 3 weeks, 3.75 g/week; KW, post hoc, *P* < .005; Table S9; symbionts: no ash vs 3 weeks, 7.5 g/week; KW, post hoc, *P* < .016; Table S9). Similarly, Zn content almost doubles in symbionts after 3 weeks of exposure to 7.5 g (Table 1). This discrete and non-systematic metal accumulation might result from a rapid biological response (e.g. hormesis-like effect) aiming at limiting toxic metal excess. This might include hyper-detoxification and/or metal overconsumption by metalloproteins (e.g. the Cu/Zn SOD). Fe and Cu both exist in multiple biologically relevant oxidation states, allowing them to accept and donate electrons. This redox activity makes Fe and Cu crucial as active centres in various proteins involved in the electron transport chain in the mitochondria and in the chloroplasts [31, 33]. In contrast, in marine environments zinc does not undergo changes in oxidation states [52], and primarily functions as a Lewis acid or in structural roles within protein complexes [83]. This redox-sensitive catalytic behaviour might explain the systematic shift observed in their isotopic compositions (Fig. 3A). Lighter Cu and to lesser extent Fe isotopic compositions (two redox-sensitive isotope proxies) are more prevalent in coral host exposed to volcanic ash, regardless of exposure duration or ash quantity (Fig. 3A), but non-redox δ^66^Zn system remains relatively stable over time. The observed difference in stable metal isotope ratio δ^66^Zn between symbionts and coral host confirms the specific and isotopically typified pattern of the organism’s reservoirs [56, 84, 85] here extending to the coral trophic chains (Table 1), but does not explain the isotopic signature in the tissue as response to the volcanic ash.

Diet and/or exogeneous pollutants like ash are expected to be the primary driver of the isotope compositions of the organism [86], although the biological processing of Cu, Zn, and Fe is expected to induce additional variabilities [49, 50]. In this study, all corals were starved meaning that diet cannot account for the observed isotopic changes. Similarly, if ash was the only and primary control of isotopic variabilities, we would expect to observe systematic Cu, Fe but also Zn isotopic variations with more pronounced changes in symbionts as they are first exposed to ash. However, this is not the case as the isotopic signature of both coral host and symbionts does not reflect the bulk ash’s isotopic signature, irrespective of intensity (amount) or duration of ash exposure (Fig. 3A). For instance, the δ^65^Cu_Coral host_ increase after 6-week ash exposure is independent of the isotopic signature in the ash, emphasizing a biologically mediated fractionation of the stable Cu isotopes rather than an accumulation of microscopic ash particles within the coral tissue over time. This inverse temporal trend, first decreasing after 3 weeks of exposure in host tissue, and then increasing after 6 weeks (Fig 3A), is also observed and even more pronounced for δ^56^Fe, suggesting that the Cu and Fe isotopic signatures observed in host tissue are not in favour of a dietary and/or ash origin but rather suggests an impaired metabolism of the redox-active bio-essential elements (i.e. Cu and Fe).

Bulk metal concentrations and isotopic ratios did not differ between pristine and leached ash (Table S7), also providing evidence that the variations observed are likely not the result of exogenous ash contamination. Nevertheless, a potential influence of ash incorporation on the isotopic signature of coral soft tissue cannot be fully excluded, as the environmental impact of ash exposure is strongly influenced by ash surface chemistry rather than its bulk composition [13]. One example is highly soluble species on the ash surface, originating through complex interactions between ash particles and volcanic gases as well as anthropogenic pollutants [87]. This impact of ash surface reactivity is observed in the lack of a positive correlation between the bulk trace metal content of ash and the amount released into the seawater [88].

For symbionts, the more stable Cu, Zn, and Fe isotopic compositions over time exposure (Fig. S1) might result from more complex and/or multiple processes (e.g. combined ash contamination and biological deregulations). This might also result from the more variable natural state, as shown by the averaged δ^65^Cu value for control symbionts [0.54‰ ± 0.15‰ (SD)], which deviates by 28% (RSD). Isotopic variabilities within symbiont samples could also be due to their dynamic life cycle. Corals frequently farm and consume their excess photosynthetic symbionts [89], with 1%–6% of symbionts degraded daily in *S. pistillata* [90], potentially fluctuating stable metal isotope ratios in the coral host tissue due to ingestion.

### Metal isotope fractionation of δ^65^Cu as an oxidative stress biomarker

As previously proposed, δ^65^Cu values in symbionts and coral hosts are indicators of stress with higher δ^65^Cu values observed in heat-stressed and bleached nubbins of *S. pistillata* [25]. In our experiments, starved non-exposed nubbins exhibit signs of stress, reflected in low effective quantum yield values of PSII (Φ_PSII_ = ∼0.3; Fig. 2) and a steady decline in Φ_PSII_ over the 6-week experimental period. Consistently, these nubbins present the highest δ^65^Cu values [0.72‰ ± 0.05‰ (SD)] within the experiment. The duration of ash exposure may affect the biological response mechanisms in lines with inversed isotopic signatures in the coral host (Fig. 3A). After an initial δ^65^Cu decrease in the coral tissue after 3 weeks of ash exposure, which aligns with signs of boosted organism, the δ^65^Cu values at 6 weeks return to values more closely associated with non-exposed corals (Fig. 3A). A similar duration-dependent effect is observed for δ^56^Fe_Coral host_, while δ^66^Zn_Coral host_ remains stable over time exposure (Fig. 3). Differences in the fractionation patterns of δ^65^Cu and δ^56^Fe compared to δ^66^Zn point to distinct enzymatic roles for these elements (Fig. 3B). As detailed earlier, the metal isotopic patterns in the coral host are not in favour of a simple ash contamination but rather suggests an impaired metabolism of the redox-active bio-essential elements (i.e. Cu and Fe).

**Figure 5.**
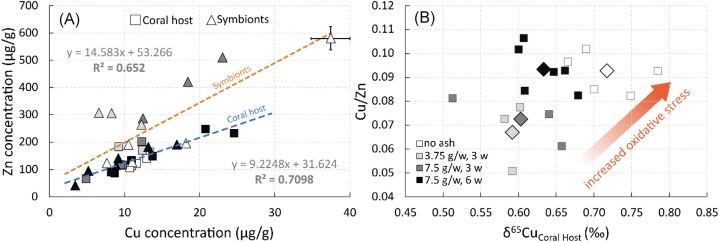
Indications of Cu/Zn SOD upregulation in *Stylophora pistillata* soft tissue. (A) Relationship between Cu and Zn concentration in the host tissue and symbionts with corresponding trendlines. (B) Influence of the Cu/Zn ratio as a biomarker for oxidative stress on δ^65^Cu_Coral host_. Data are presented as the mean (◇) of individual measurements in the host tissue (□). The white-to-black colour gradient resembles the intensity of ash exposure.

When plotting Cu and Zn concentrations in symbionts and in tissue, a significant positive correlation (symbionts: *R*^2 ^= 0.65, *P* < .001; coral host: *R*^2 ^= 0.71, *P* < .001; Fig. 5a; Table S10) with similar positive slopes (Welch, *P* = .11; Fig. 5a) can be observed. This suggests that Cu and Zn homeostasis might be regulated by a similar mechanism, such as the expression of Cu/Zn SOD, in which Zn and Cu are major structural and catalytic cofactors. SOD is a key enzyme for scavenging ROS in symbiotic corals [43, 44]. In mammals, increased SOD expression enhances the resistance to oxidative damage [91]. SOD upregulation is associated with decreasing Cu/Zn ratios [92], which serves as a biomarker for inflammation and/or infection [93], with high values of Cu/Zn linked to increased oxidative stress [94, 95]. In this study, we observed that an increase in the Cu/Zn ratio aligns positively with higher δ^65^Cu_Coral host_ (Fig. 5b), suggesting that δ^65^Cu_Coral host_ may reflect changes in the antioxidant capacity and/or ROS production within the holobiont. We propose that in response to enhanced ROS production due to increased photosynthetic activity driven by alleviated micronutrient limitations, the coral host first up-regulates Cu/Zn SOD to prevent oxidative damage. Although the relationship of Cu/Zn ratio and the Cu/Zn SOD activity in the coral soft tissue needs to be experimentally evaluated, monitoring and analysing redox-sensitive isotopic proxies such as δ^65^Cu_Coral host_ provides valuable insights into the photophysiological state of the coral holobiont. Further research is needed to better constrain the relationship between Cu isotopic fractionation with symbiont’s photosynthetic processes, especially in antioxidant defence mechanisms.

The increase in δ^65^Cu_Coral host_ after 6 weeks of ash exposure corresponds with elevated Cu/Zn values (Fig. 5b), and indicates increased stress levels compared to the 3-week conditions. These inverse patterns suggest harmful effect over longer-term (i.e. chronic) exposure. As molecular bonds with lighter isotopes are generally weaker than those with heavier isotopes [96], they are more susceptible to disruption by ROS damage. This disruption and subsequent release from the coral host can lead to heavier isotopic signatures as seen in both the control and 6-week ash exposure conditions (Fig. 3). Increased stress and/or a decrease in the antioxidant defence mechanisms in the non-exposed and 6-week ash-exposed corals may be attributed to the prolonged starvation in both cases. Furthermore, the continuous addition of pristine and fertile volcanic ash can, at the end, induce toxicity, which leads to metal enhanced ROS production [40]. Under such stress conditions, ROS produced by endosymbionts can cause photodamage to PSII and decrease the maximum quantum yield of PSII in both corals and free-living *Symbiodinium* sp. [97]. However, in our experiments, no such photodamage was observed after 6 weeks of ash exposure. Instead, a significant increase in F_v_/F_m_ (WMW, *P* < .003; Table S3) highlights the beneficial effect of ash exposure on the coral organism. Furthermore, no significant change in the ROS-induced lipid peroxidation was detected in *S. pistillata* in the 6-week ash-exposed coral compared to the control references [22]. This could suggest that redox-sensitive Cu isotopic variations appear upstream of biological alterations. Therefore, we propose that δ^65^Cu_Coral host_ reveals even minor changes in the coral’s physiological condition and that δ^65^Cu has the potential to monitor the accumulation of stress, which makes it a sensitive biomarker for coral stress. This sensitivity may explain the significant inter- and intracolony variations observed in δ^65^Cu in coral skeletons, independent of seawater composition [59]. Evidence suggests that the isotopic signature in coral skeleton is linked to the distribution of isotopes within coral tissues. For instance, heat stress increased the Cu, and to a lesser extent the Zn isotopic composition in coral tissue [25], whereas an increase in temperature generally results in a decrease of δ^66^Zn in *Porites* sp. skeleton [58]. If a connection exists between δ^65^Cu in coral tissue and the skeleton, sampling the skeleton could reveal significant insights into the coral’s physiological state throughout itsonmental stress. J Toxicol Environ Healt lifespan.

Understanding the underlying causes of metal isotope fractionation and their partitioning among various coral compartments (symbionts, coral host, and skeleton) will provide valuable insights into micronutrient cycling within the coral holobiont. Establishing a direct link between redox-sensitive isotopic systems, such as δ^56^Fe and δ^65^Cu, in the hard coral tissue, could serve as a powerful tool for assessing their physiological state. This approach would not only improve our understanding of micronutrient management in modern corals under varying environmental conditions, but also open up the possibility of using fossilized corals as paleoenvironmental proxies to infer past coral health.

## Conclusion

Volcanic ash from the La Soufrière, St. Vincent eruption, provides essential micronutrients that enhance photochemical processes within the coral holobiont, as indicated by improved photosynthetic parameters (e.g. F_v_/F_m_, rETR, NPQ, and net photosynthesis rates). The quantity and duration of ash exposure appear to play a subordinate role as even a reduced ash supply significantly boosts the effective quantum yield of photosystem II (Φ_PSII_), suggesting that certain metal thresholds can be met with even lower ash levels. Although Fe, Cu, and Zn concentrations in coral host and symbionts show no systematic difference between ash-exposed and control corals, more systematic shifts in the δ^56^Fe and δ^65^Cu redox-sensitive isotopic proxies were observed. The shifts in δ^65^Cu in ash-exposed coral tissues, although not statistically significant (except for control vs exposed over 3 weeks at 3.75 g/week), strongly correlate with enhanced photosynthetic parameters, suggesting that Cu isotope fractionation may reflect changes in the symbiont’s photophysiological performance, with lighter δ^65^Cu linked to more efficient photosynthetic activity. However, the beneficial effects of ash exposure appear time-sensitive, as prolonged exposure could disrupt metal homeostasis as marked by inversed redox-sensitive isotopic patterns, potentially increasing the risk of metal toxicity. We propose that δ^65^Cu in coral hosts may serve as a biomarker for assessing the physiological state of symbiotic corals, with an intricate link between δ^65^Cu and the antioxidant defences of the coral holobiont. Further research is needed to fully establish the relationship between Cu isotopic fractionation in the coral host and the photosynthetic processes of its symbionts. Our findings emphasize the dynamic response of coral tissue’s isotopic signatures to environmental changes, such as micronutrient cycling from volcanic aerosols. Understanding these relationships will advance our knowledge of coral resilience mechanisms in response to natural stressors.

## Supplementary Material

mfaf011_Supplemental_File

## Data Availability

The original data that support the findings of this study are available in Mendeley Data with the identifier doi:10.17632/924gz5795p.2
