# Supplementary material for: Redox-sensitive δ65Cu isotopic fractionation in the tissue of the scleractinian coral Stylophora pistillata: a biomarker of holobiont photophysiology following volcanic ash exposure
Source: Metallomics. 2025 Apr 23;17(5):mfaf011. doi: 10.1093/mtomcs/mfaf011 (PMC12050973; doi:10.1093/mtomcs/mfaf011)
Supplement: mfaf011_Supplemental_File [file mfaf011_supplemental_file.docx]

**Supplementary Material for “δ^65^Cu isotopic fractionation in coral tissue*:* A biomarker of holobiont photophysiology following volcanic ash exposure*” (Förster et al., 2025)***


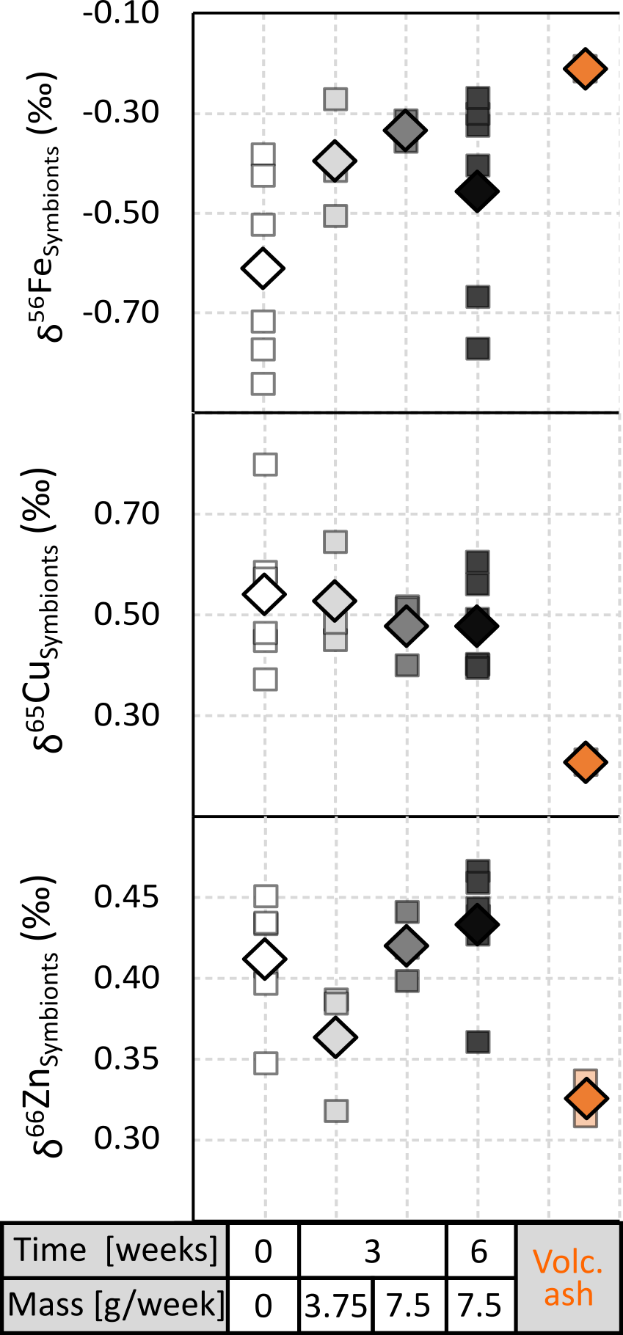


Fig. S1: Metal stable isotope ratios δ^56^Fe, δ^65^Cu and δ^66^Zn in the symbionts of *Stylophora pistillata* maintained under four ash exposure conditions. Data is presented as the mean (◇) of individual measurements of symbionts (□) and the averaged values for pristine and leached volcanic ash. The white-to-black colour gradient resembles the intensity of ash exposure.


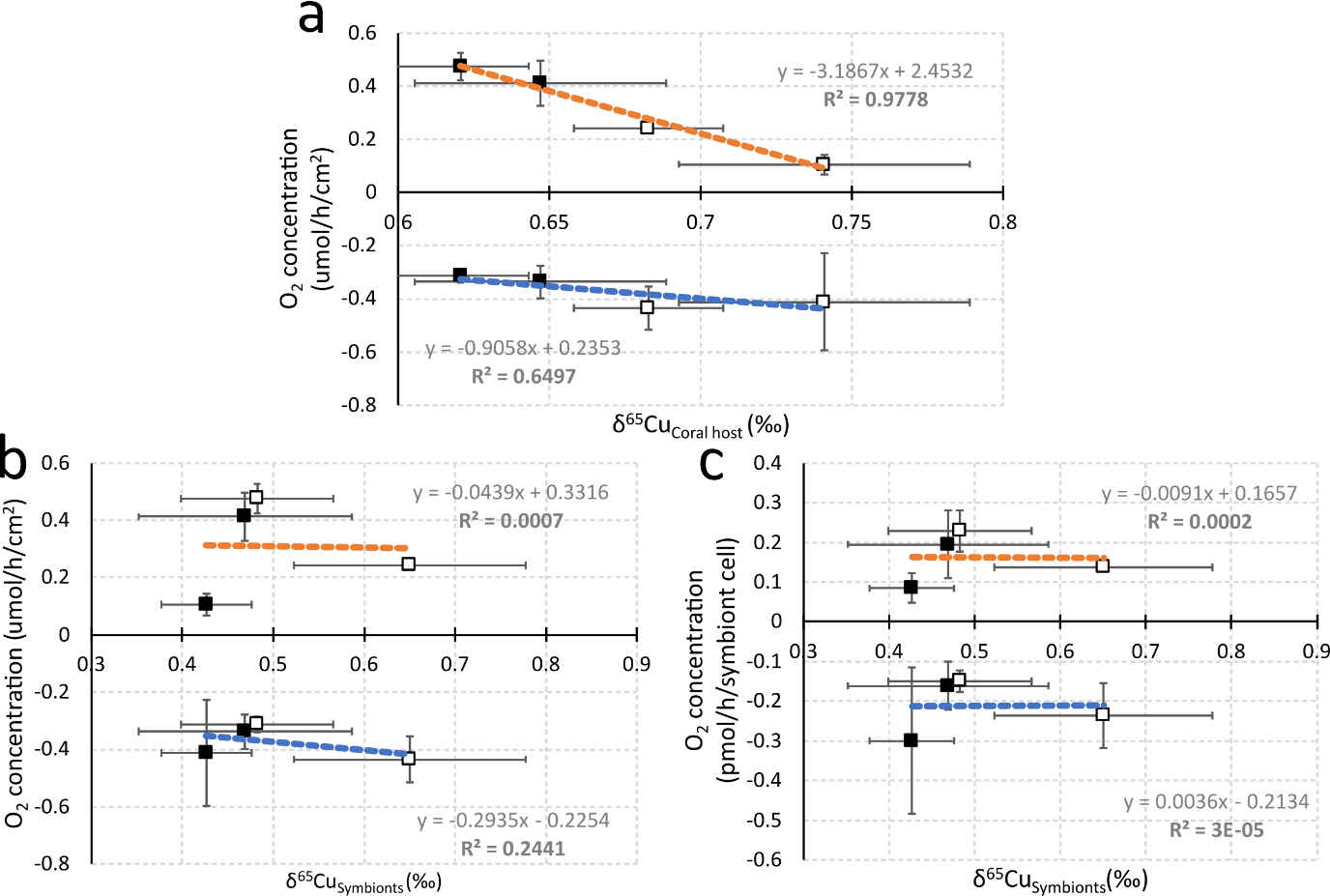


Fig. S2: Relationship between δ^65^Cu in *Stylophora pistillata* with the net photosynthesis and respiration rates of non-exposed and six-week ash exposed nubbins. (a) δ^65^Cu_Coral host_ vs. change in oxygen concentration per hour normalized per surface area. (b) δ^65^Cu_Symbionts_ vs change in oxygen concentration per hour normalized per surface area and per symbiont cell. Each point represents the mean oxygen production/consumption per tank (n = 3). Data is presented as mean ± SD.


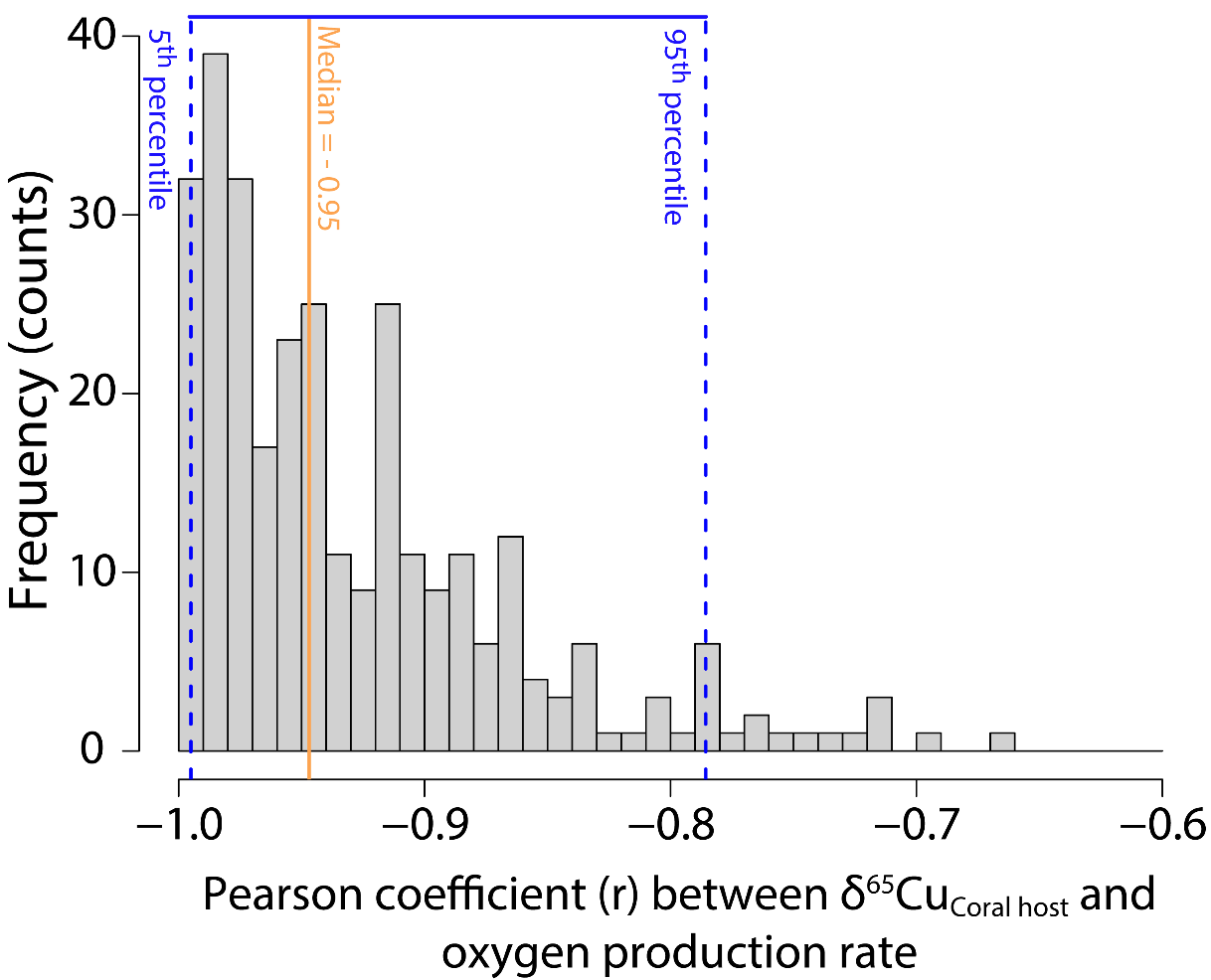


Fig. S3: Histogram of calculated Pearson correlation coefficients (r) between bootstrapped raw data of δ^65^Cu_Coral host_ and oxygen production rate, normalized per symbiont cell. The data consist of resampled mean values for δ^65^Cu_Coral host_ and oxygen production rate from each experimental tank, with r calculated for each resampling. The resampling procedure was repeated 300 times. Blue dotted lines present the 90 % credible range for r (5^th^ and 95^th^ quantile), while the orange solid line represents the 50^th^ quantile (median).

Table S1: Long-term external reproducibility (bracketing standards) of δ^65^Cu, δ^66^Zn, δ^56^Fe measurements

| Sample Name  IRMM524 | δ^56^Fe  IRMM14  (‰) | Sample Name NBS976 | δ^65^Cu  NBS976  (‰) | Sample Name JMC-Lyon | δ^66^Zn  JMC-Lyon  (‰) |
| --- | --- | --- | --- | --- | --- |
|  |  |  |  |  |  |
| IRMM524_1ppm | -0.04 | Std_Cu/Zn_NBS/AEASAR100ppb | -0.09 | Std_Cu/Zn_NBS/AEASAR250ppb | 0.31 |
| IRMM524_1ppm | 0.03 | Std_Cu/Zn_NBS/AEASAR100ppb | -0.05 | Std_Cu/Zn_NBS/AEASAR250ppb | 0.29 |
| IRMM524_1ppm | 0.11 | Std_Cu/Zn_NBS/AEASAR100ppb | 0.00 | Std_Cu/Zn_NBS/AEASAR250ppb | 0.31 |
| IRMM524_1ppm | -0.18 | Std_Cu/Zn_NBS/AEASAR100ppb | 0.01 | Std_Cu/Zn_NBS/AEASAR250ppb | 0.33 |
| IRMM524_1ppm | 0.01 | Std_Cu/Zn_NBS/AEASAR100ppb | -0.04 | Std_Cu/Zn_NBS/AEASAR250ppb | 0.21 |
| IRMM524_1ppm | -0.01 | Std_Cu/Zn_NBS/AEASAR100ppb | -0.02 | Std_Cu/Zn_NBS/AEASAR250ppb | 0.26 |
| IRMM524_1ppm | 0.09 | Std_Cu/Zn_NBS/AEASAR100ppb | -0.03 | Std_Cu/Zn_NBS/AEASAR250ppb | 0.27 |
| IRMM524_1ppm | -0.05 | Std_Cu/Zn_NBS/AEASAR100ppb | 0.00 | Std_Cu/Zn_NBS/AEASAR250ppb | 0.30 |
| IRMM524_1ppm | 0.02 | Std_Cu/Zn_NBS/AEASAR100ppb | 0.01 | Std_Cu/Zn_NBS/AEASAR250ppb | 0.26 |
| IRMM524_1ppm | 0.01 | Std_Cu/Zn_NBS/AEASAR100ppb | 0.04 | Std_Cu/Zn_NBS/AEASAR250ppb | 0.27 |
| IRMM524_1ppm | -0.06 | Std_Cu/Zn_NBS/AEASAR100ppb | -0.01 | Std_Cu/Zn_NBS/AEASAR250ppb | 0.12 |
| IRMM524_1ppm | 0.04 | Std_Cu/Zn_NBS/AEASAR100ppb | 0.02 | Std_Cu/Zn_NBS/AEASAR250ppb | 0.22 |
| IRMM524_1ppm | 0.16 | Std_Cu/Zn_NBS/AEASAR100ppb | -0.02 | Std_Cu/Zn_NBS/AEASAR250ppb | 0.32 |
| IRMM524_1ppm | -0.13 | Std_Cu/Zn_NBS/AEASAR100ppb | -0.08 | Std_Cu/Zn_NBS/AEASAR250ppb | 0.24 |
| IRMM524_1ppm | -0.02 | Std_Cu/Zn_NBS/AEASAR100ppb | -0.06 | Std_Cu/Zn_NBS/AEASAR250ppb | 0.28 |
| IRMM524_1ppm | -0.06 | Std_Cu/Zn_NBS/AEASAR100ppb | -0.05 | Std_Cu/Zn_NBS/AEASAR250ppb | 0.27 |
| IRMM524_1ppm | 0.00 | Std_Cu/Zn_NBS/AEASAR100ppb | -0.02 | Std_Cu/Zn_NBS/AEASAR250ppb | 0.29 |
| IRMM524_1ppm | 0.06 | Std_Cu/Zn_NBS/AEASAR100ppb | 0.00 | Std_Cu/Zn_NBS/AEASAR250ppb | 0.29 |
| IRMM524_1ppm | -0.07 | Std_Cu/Zn_NBS/AEASAR100ppb | 0.01 | Std_Cu/Zn_NBS/AEASAR250ppb | 0.27 |
| IRMM524_1ppm | 0.04 | Std_Cu/Zn_NBS/AEASAR100ppb | -0.06 | Std_Cu/Zn_NBS/AEASAR250ppb | 0.42 |
| IRMM524_1ppm | 0.04 | Std_Cu/Zn_NBS/AEASAR100ppb | -0.06 | Std_Cu/Zn_NBS/AEASAR250ppb | 0.25 |
| IRMM524_1ppm | 0.06 | Std_Cu/Zn_NBS/AEASAR100ppb | 0.04 | Std_Cu/Zn_NBS/AEASAR250ppb | 0.18 |
| IRMM524_1ppm | -0.14 | Std_Cu/Zn_NBS/AEASAR100ppb | -0.04 | Std_Cu/Zn_NBS/AEASAR250ppb | 0.21 |
| IRMM524_1ppm | 0.01 | Std_Cu/Zn_NBS/AEASAR100ppb | -0.02 | Std_Cu/Zn_NBS/AEASAR250ppb | 0.38 |
| IRMM524_1ppm | 0.17 | Std_Cu/Zn_NBS/AEASAR100ppb | -0.05 | Std_Cu/Zn_NBS/AEASAR250ppb | 0.27 |
| IRMM524_1ppm | -0.14 | Std_Cu/Zn_NBS/AEASAR100ppb | 0.01 | Std_Cu/Zn_NBS/AEASAR250ppb | 0.30 |
| IRMM524_1ppm | 0.10 | Std_Cu/Zn_NBS/AEASAR100ppb | 0.08 | Std_Cu/Zn_NBS/AEASAR250ppb | 0.26 |
| IRMM524_1ppm | -0.13 | Std_Cu/Zn_NBS/AEASAR100ppb | 0.01 | Std_Cu/Zn_NBS/AEASAR250ppb | 0.28 |
| IRMM524_1ppm | 0.00 | Std_Cu/Zn_NBS/AEASAR100ppb | -0.03 | Std_Cu/Zn_NBS/AEASAR250ppb | 0.29 |
| IRMM524_1ppm | 0.11 | Std_Cu/Zn_NBS/AEASAR100ppb | -0.03 | Std_Cu/Zn_NBS/AEASAR250ppb | 0.27 |
| IRMM524_1ppm | 0.02 | Std_Cu/Zn_NBS/AEASAR100ppb | 0.10 | Std_Cu/Zn_NBS/AEASAR250ppb | 0.28 |
| IRMM524_1ppm | 0.02 | Std_Cu/Zn_NBS/AEASAR100ppb | -0.01 | Std_Cu/Zn_NBS/AEASAR250ppb | 0.24 |
| IRMM524_1ppm | -0.04 | Std_Cu/Zn_NBS/AEASAR100ppb | -0.08 | Std_Cu/Zn_NBS/AEASAR250ppb | 0.28 |
| IRMM524_1ppm | 0.01 | Std_Cu/Zn_NBS/AEASAR100ppb | 0.02 | Std_Cu/Zn_NBS/AEASAR250ppb | 0.24 |
| IRMM524_1ppm | 0.14 | Std_Cu/Zn_NBS/AEASAR100ppb | 0.06 | Std_Cu/Zn_NBS/AEASAR250ppb | 0.33 |
| IRMM524_1ppm | -0.16 | Std_Cu/Zn_NBS/AEASAR100ppb | 0.02 | Std_Cu/Zn_NBS/AEASAR250ppb | 0.27 |
| IRMM524_1ppm | 0.16 | Std_Cu/Zn_NBS/AEASAR100ppb | -0.05 | Std_Cu/Zn_NBS/AEASAR250ppb | 0.29 |
| IRMM524_1ppm | 0.13 | Std_Cu/Zn_NBS/AEASAR100ppb | 0.11 | Std_Cu/Zn_NBS/AEASAR250ppb | 0.18 |
| IRMM524_1ppm | 0.01 | Std_Cu/Zn_NBS/AEASAR100ppb | -0.03 | Std_Cu/Zn_NBS/AEASAR250ppb | 0.25 |
| IRMM524_1ppm | -0.11 | Std_Cu/Zn_NBS/AEASAR100ppb | -0.06 | Std_Cu/Zn_NBS/AEASAR250ppb | 0.31 |
| IRMM524_1ppm | 0.12 | Std_Cu/Zn_NBS/AEASAR100ppb | -0.08 |  |  |
| IRMM524_1ppm | -0.03 | Std_Cu/Zn_NBS/AEASAR100ppb | 0.09 |  |  |
| IRMM524_1ppm | -0.02 | Std_Cu/Zn_NBS/AEASAR100ppb | -0.08 |  |  |
| IRMM524_1ppm | 0.00 | Std_Cu/Zn_NBS/AEASAR100ppb | -0.11 |  |  |
| IRMM524_1ppm | 0.02 |  |  |  |  |
| IRMM524_1ppm | 0.01 |  |  |  |  |
| IRMM524_1ppm | -0.01 |  |  |  |  |
| IRMM524_1ppm | 0.03 |  |  |  |  |
| IRMM524_1ppm | -0.06 |  |  |  |  |
| IRMM524_1ppm | 0.17 |  |  |  |  |
| IRMM524_1ppm | 0.14 |  |  |  |  |
| IRMM524_1ppm | 0.05 |  |  |  |  |
| IRMM524_1ppm | 0.02 |  |  |  |  |
| IRMM524_1ppm | -0.01 |  |  |  |  |
| IRMM524_1ppm | -0.04 |  |  |  |  |
| IRMM524_1ppm | 0.11 |  |  |  |  |
| IRMM524_1ppm | -0.08 |  |  |  |  |
| IRMM524_1ppm | 0.06 |  |  |  |  |
| IRMM524_1ppm | 0.04 |  |  |  |  |
| IRMM524_1ppm | -0.03 |  |  |  |  |
| IRMM524_1ppm | -0.02 |  |  |  |  |
| IRMM524_1ppm | 0.09 |  |  |  |  |
| IRMM524_1ppm | -0.12 |  |  |  |  |
| IRMM524_1ppm | 0.03 |  |  |  |  |
| IRMM524_1ppm | 0.11 |  |  |  |  |
| IRMM524_1ppm | -0.18 |  |  |  |  |
| External repro  d56Fe (2sd, n=80) | **0.17** | **External repro  d65Cu (2sd, n=43)** | **0.10** | **External repro  d66Zn (2sd, n=40)** | **0.10** |

Table S2: Statistical results of Φ_PSII_ measurements on nubbins of the coral *Stylophora pistillata* between tanks under identical condition.

Kruskal-Wallis test (KW) and Dunn’s post-hoc test, with Holm-Bonferroni correction for p-value adjustment was performed to identify statistically significant differences between tanks of the same condition (**bold**) when p ≤ 0.05.

| **Comparison** | |  | **Aquaria** | | z-value | adj. p-value |
| --- | --- | --- | --- | --- | --- | --- |
| *Condition 1* | *Condition 2* |  | *Aquaria 1* | *Aquaria 2* |  |  |
| no ash | no ash |  | A | B | -0.382 | 0.702 |
| 3 weeks, 7.5g | 6 weeks, 7.5g |  | D | E | 1.543 | 0.306 |
| 3 weeks, 7.5g | 6 weeks, 7.5g |  | D | F | -0.132 | 0.447 |
| 6 weeks, 7.5g | 6 weeks, 7.5g |  | E | F | 1.453 | 0.293 |

*A & B - no ash; C - 3 weeks, 3.75g; D - 3 weeks, 7.5g; E & F - 6 weeks, 7.5 g*

Table S3: Statistical results of the effect of volcanic ash on the maximum quantum yield of PSII (F_v_/F_m_) of the coral *Stylophora pistillata* with increasing PAR-values.
Normal distribution and homoskedasticity were assessed using Shapiro-Wilk test and Levene test, respectively. Based on former and latter outcome, statistical tests (ANOVA, WMW and Welch) were performed. Differences between control and ash-exposed condition were termed statistically significant when p ≤ 0.05 in all cases. Levels of significance are indicated as p-value > 0.05 (not significant), p-value ≤ 0.05 (*), p-value ≤ 0.01 (**), and p-value ≤ 0.001 (***).

| **PAR** | **n** | **Normality** | | **Homoskedacity** | | **Statistical test** | | |
| --- | --- | --- | --- | --- | --- | --- | --- | --- |
|  |  | *W* | *p-value* | *F* | *p-value* | *Type* | *p-value* | *Level of significance* |
| 0 | 6 | 0.793 | **0.008** | 0.036 | 0.853 | WMW | **0.002** | ** |
| 10 | 6 | 0.778 | **0.005** | 3.827 | 0.079 | WMW | **0.002** | ** |
| 17 | 6 | 0.768 | **0.004** | 3.481 | 0.092 | WMW | **0.002** | ** |
| 26 | 6 | 0.765 | **0.004** | 4.994 | 0.049 | WMW | **0.002** | ** |
| 41 | 6 | 0.780 | **0.006** | 0.891 | 0.367 | WMW | **0.005** | ** |
| 74 | 6 | 0.818 | **0.015** | 0.182 | 0.679 | WMW | **0.002** | ** |
| 130 | 6 | 0.878 | 0.084 | 1.485 | 0.251 | ANOVA | **3.66E-06** | *** |
| 220 | 6 | 0.916 | 0.255 | 3.186 | 0.105 | ANOVA | **3.25E-04** | *** |
| 664 | 6 | 0.961 | 0.803 | 1.110 | 0.317 | ANOVA | 0.906 |  |
| 1032 | 6 | 0.962 | 0.806 | 0.378 | 0.553 | ANOVA | **0.041** | * |
| 1956 | 6 | 0.978 | 0.976 | 0.287 | 0.604 | ANOVA | **0.015** | * |

Table S4: Statistical results of the effect of volcanic ash on the relative electron transport rate (rETR) with increasing PAR-values.
Normal distribution and homoskedasticity were assessed using Shapiro-Wilk test and Levene test, respectively. Based on former and latter outcome, statistical tests (ANOVA, WMW and Welch) were performed. Differences between control and ash-exposed condition were termed statistically significant when p ≤ 0.05 in all cases. Levels of significance are indicated as p-value > 0.05 (not significant), p-value ≤ 0.05 (*), p-value ≤ 0.01 (**), and p-value ≤ 0.001 (***).

| **PAR** | **n** | **Normality** | | **Homoskedacity** | | **Statistical test** | | |
| --- | --- | --- | --- | --- | --- | --- | --- | --- |
|  |  | *W* | *p-value* | *F* | *p-value* | *Type* | *p-value* | *Level of significance* |
| 0 | 6 | - | - | - | - | - | - | - |
| 10 | 6 | 0.770 | **0.004** | 8.929 | 0.014 | WMW | **0.004** | ** |
| 17 | 6 | 0.769 | **0.004** | 2.222 | 0.167 | WMW | **0.004** | ** |
| 26 | 6 | 0.767 | **0.004** | 4.364 | 0.063 | WMW | **0.004** | ** |
| 41 | 6 | 0.782 | **0.006** | 0.870 | 0.373 | WMW | **0.005** | ** |
| 74 | 6 | 0.823 | **0.018** | 0.242 | 0.634 | WMW | **0.002** | ** |
| 130 | 6 | 0.880 | 0.088 | 1.450 | 0.256 | ANOVA | **0.000** | *** |
| 220 | 6 | 0.917 | 0.261 | 3.143 | 0.107 | ANOVA | **0.000** | *** |
| 664 | 6 | 0.964 | 0.833 | 1.205 | 0.298 | ANOVA | 0.895 |  |
| 1032 | 6 | 0.963 | 0.823 | 0.376 | 0.553 | ANOVA | **0.038** | * |
| 1956 | 6 | 0.979 | 0.980 | 0.332 | 0.577 | ANOVA | **0.014** | * |

Table S5: Statistical results of the effect of volcanic ash on the non-photochemical quenching (NPQ) of the coral *Stylophora pistillata* with increasing PAR-values.
Normal distribution and homoskedasticity were assessed using Shapiro-Wilk test and Levene test, respectively. Based on former and latter outcome, statistical tests (ANOVA, WMW and Welch) were performed. Differences between control and ash-exposed condition were termed statistically significant when p ≤ 0.05 in all cases. Levels of significance are indicated as p-value > 0.05 (not significant), p-value ≤ 0.05 (*), p-value ≤ 0.01 (**), and p-value ≤ 0.001 (***).

| **PAR** | **n** | **Normality** | | **Homoskedacity** | | **Statistical test** | | |
| --- | --- | --- | --- | --- | --- | --- | --- | --- |
|  |  | *W* | *p-value* | *F* | *p-value* | *Type* | *p-value* | *Level of significance* |
| 0 | 6 | - | - | - | - | - | - | - |
| 10 | 6 | 0.891 | 0.120 | 1.500 | 0.249 | ANOVA | 0.066 |  |
| 17 | 6 | 0.923 | 0.316 | 0.007 | 0.933 | ANOVA | 0.552 |  |
| 26 | 6 | 0.925 | 0.333 | 0.761 | 0.404 | ANOVA | **0.010** | * |
| 41 | 6 | 0.923 | 0.312 | 0.718 | 0.417 | ANOVA | **0.001** | ** |
| 74 | 6 | 0.918 | 0.267 | 1.104 | 0.318 | ANOVA | **4.51E-04** | *** |
| 130 | 6 | 0.920 | 0.287 | 1.467 | 0.254 | ANOVA | **1.69E-04** | *** |
| 220 | 6 | 0.916 | 0.256 | 1.662 | 0.226 | ANOVA | **6.19E-05** | *** |
| 664 | 6 | 0.909 | 0.209 | 1.522 | 0.246 | ANOVA | **3.14E-05** | *** |
| 1032 | 6 | 0.902 | 0.167 | 1.356 | 0.271 | ANOVA | **1.84E-05** | *** |
| 1956 | 6 | 0.899 | 0.154 | 1.465 | 0.254 | ANOVA | **1.58E-05** | *** |

Table S6: Statistical results of the effect of volcanic ash on net photosynthesis and respiration rates of the coral *Stylophora pistillata.*
Normal distribution and homoskedasticity were assessed using Shapiro-Wilk test and Levene test, respectively. Based on former and latter outcome, statistical tests (ANOVA, WMW and Welch) were performed. Differences between control and ash-exposed condition were termed statistically significant when p ≤ 0.05 in all cases. Levels of significance are indicated as p-value > 0.05 (not significant), p-value ≤ 0.05 (*), p-value ≤ 0.01 (**), and p-value ≤ 0.001 (***).

| **Measurement** |  | **Normality** | | **Homoskedasticity** | | **Statistical test** | | |
| --- | --- | --- | --- | --- | --- | --- | --- | --- |
|  | ***n*** | *W* | *p-value* | *F* | *p-value* | *Type* | *p-value* | *level of significance* |
| Net photosynthesis rate per skeletal surface area | 12 | 0.933 | 0.416 | 0.817 | 0.387 | ANOVA | **9.75E-05** | *** |
| Net photosynthesis rate per symbiont cell | 12 | 0.957 | 0.744 | 0.002 | 0.963 | ANOVA | **0.003** | ** |
| Net respiration rate per skeletal surface area | 12 | 0.838 | **0.027** | 3.693 | 0.084 | WMW | 0.200 |  |
| Net respiration rate per symbiont cell | 12 | 0.869 | 0.063 | 9.995 | **0.010** | Welch | **0.010** | * |

Table S7: Chemical characterization of pristine (p) volcanic ash and leached (l) volcanic ash samples, gathered on Barbados (B). Minor and trace elements were measured on an ICP-MS, and major elements on an ICP-OES. Data is presented as total concentration whether as ppm for minor and trace elements or as wt.% oxide for major elements.

| **a) Trace metals** | **pB.a** | **pB.b** | **lB.a** | **lB.b** | **BHVO2.a** | **BHVO2.b** | **DR-N** | **BHVO-1** |
| --- | --- | --- | --- | --- | --- | --- | --- | --- |
|  | *[µg/g]* | *[µg/g]* | *[µg/g]* | *[µg/g]* | *[µg/g]* | *[µg/g]* | *[µg/g]* | *[µg/g]* |
| V | 179.04 | 151.17 | 187.47 | 168.46 | 357.90 | 333.06 | - | - |
| Cr | 26.53 | 23.07 | 28.47 | 25.12 | 311.79 | 292.57 | - | - |
| Co | 17.21 | 14.59 | 17.88 | 16.57 | 47.76 | 45.06 | - | - |
| Ni | 15.59 | 13.44 | 15.74 | 14.35 | 127.06 | 120.44 | - | - |
| Cu | 125.37 | 107.32 | 133.68 | 124.14 | 136.24 | 127.31 | - | - |
| Zn | 267.29 | 229.53 | 318.26 | 290.29 | 116.59 | 107.51 | - | - |
| Ga | 22.06 | 18.92 | 22.98 | 21.30 | 24.24 | 22.10 | - | - |
| As | 2.82 | 2.67 | 2.91 | 2.79 | 1.59 | 1.61 | - | - |
| Se | 0.35 | 0.35 | 0.24 | 0.26 | 0.14 | 0.17 | - | - |
| Rb | 13.66 | 14.09 | 14.45 | 14.47 | 8.13 | 8.75 | - | - |
| Sr | 204.21 | 199.86 | 222.07 | 211.06 | 361.46 | 362.96 | - | - |
| Mo | <l.o.d | <l.o.d | <l.o.d | <l.o.d | 2.92 | 3.39 | - | - |
| Cd | 0.19 | 0.20 | 0.19 | 0.20 | 0.17 | 0.29 | - | - |
| Ba | 129.54 | 128.49 | 139.18 | 133.63 | 119.23 | 121.88 | - | - |
| Ce | 14.97 | 15.11 | 16.09 | 15.63 | 33.51 | 34.59 | - | - |
| Pb | 2.79 | 3.00 | 3.06 | 3.19 | 1.40 | 1.67 | - | - |
| **b) Isotope ratios** | *[%_0_]* | *[%_0_]* | *[%_0_]* | *[%_0_]* | *[%_0_]* | *[%_0_]* |  |  |
| δ^56^Fe | -0.24 | -0.18 | -0.21 | -0.22 | 0.03 | 0.00 | - | - |
| δ^65^Cu | 0.20 | 0.27 | 0.21 | 0.27 | 0.12 | 0.24 | - | - |
| δ^66^Zn | 0.31 | - | 0.34 | - | 0.24 | 0.20 | - | - |
| **c) Oxides** | *[wt.%]* | *[wt.%]* | *[wt.%]* | *[wt.%]* | *[wt.%]* | *[wt.%]* | *[wt.%]* | *[wt.%]* |
| SiO_2_ | 56.11 | 55.62 | 55.82 | 55.56 | 50.13 | - | 53.05 | 49.93 |
| Al_2_O_3_ | 19.15 | 19.06 | 19.21 | 19.04 | 13.68 | - | 17.74 | 13.77 |
| Fe_2_O_3_ | 7.39 | 7.62 | 7.24 | 7.21 | 12.62 | - | 9.76 | 12.53 |
| MgO | 2.91 | 2.90 | 2.86 | 2.87 | 7.27 | - | 4.29 | 7.22 |
| CaO | 8.04 | 8.00 | 8.08 | 8.01 | 11.38 | - | 6.99 | 11.35 |
| Na2O | 3.83 | 3.79 | 3.87 | 3.85 | 2.26 | - | 2.99 | 2.30 |
| K_2_O | 0.64 | 0.63 | 0.64 | 0.64 | 0.50 | - | 1.69 | 0.51 |
| TiO_2_ | 0.79 | 0.79 | 0.78 | 0.78 | 2.69 | - | 1.04 | 2.68 |
| MnO | 0.15 | 0.15 | 0.15 | 0.15 | 0.17 | - | 0.21 | 0.17 |
| P_2_O_5_ | 0.15 | 0.16 | 0.15 | 0.15 | 0.27 | - | 0.23 | 0.28 |
| Ba | 0.01 | 0.01 | 0.01 | 0.01 | 0.01 | - | 0.04 | 0.01 |
| Sr | 0.02 | 0.02 | 0.02 | 0.02 | 0.04 | - | 0.04 | 0.04 |
| H_2_O^+^ | 0.45 | 0.45 | - | - | 0.18 | - | 2.22 | 0.16 |
| H_2_O^-^ | 0.24 | 0.24 | - | - | 0 | - | 0.25 | 0.05 |
| **Total** | 99.88 | 99.44 | 98.83 | 98.29 | 100.50 | - | 100.51 | 100.98 |

Table S8: Statistical results of Fe, Cu, and Zn concentrations and their isotope ratios in the coral host tissue compared to its symbionts in the coral *Stylophora pistillata.*

Kruskal-Wallis test (KW) and Dunn’s post-hoc test, with Holm-Bonferroni correction for p-value adjustment was performed to identify statistically significant differences between the metal concentration in coral host and its symbionts (**bold**) when p ≤ 0.05.

| **Condition** | **Sample size** |  | **Fe** | **Cu** | **Zn** | **δ^56^Fe** | **δ^65^Cu** | **δ^66^Zn** |
| --- | --- | --- | --- | --- | --- | --- | --- | --- |
| no ash | 6 | *z-score* | -0.951 | -0.055 | -1.520 | 1.397 | 2.922 | -3.398 |
|  |  | *adj. p-value* | 1.000 | 1.000 | 1.000 | 1.000 | **0.045** | **0.009** |
| 3 weeks, 3.75g/week | 3 | *z-score* | -0.328 | 0.451 | -1.722 | -1.744 | 0.518 | -0.736 |
|  |  | *adj. p-value* | 1.000 | 1.000 | 0.808 | 0.812 | 1.000 | 1.000 |
| 3 weeks, 7.5g/week | 3 | *z-score* | -2.419 | -2.050 | -2.378 | -2.480 | 1.434 | -1.434 |
|  |  | *adj. p-value* | 0.187 | 0.525 | 0.218 | 0.171 | 1.000 | 1.000 |
| 6 weeks, 7.5g/week | 6 | *z-score* | -0.735 | 1.072 | 0.276 | -0.219 | 2.592 | -2.795 |
|  |  | *adj. p-value* | 1.000 | 1.000 | 1.000 | 1.000 | 0.119 | 0.065 |

Table S9: Statistical results of Fe, Cu, and Zn concentrations and their isotope ratios in the coral host tissue and symbionts in the coral *Stylophora pistillata.*

Kruskal-Wallis test (KW) and Dunn’s post-hoc test, with Holm-Bonferroni correction for p-value adjustment was performed to identify statistically significant differences between the metal concentration in coral host and its symbionts (**bold**) when p ≤ 0.05.

|  | **Comparison** | | **Coral Host** | | **Symbionts** | |
| --- | --- | --- | --- | --- | --- | --- |
|  | *Condition 1* | *Condition 2* | *z-value* | *adjusted* | *z-value* | *adjusted* |
|  |  |  |  | *p-value^1^* |  | *p-value^1^* |
| **Fe** | no ash | 3 weeks, 3.75 g | 3.179 | **0.004** | 1.285 | 0.298 |
|  | no ash | 3 weeks, 7.5 g | 1.148 | 0.251 | 2.819 | **0.015** |
|  | no ash | 6 weeks, 7.5 g | 1.730 | 0.125 | 0.664 | 0.253 |
|  | 3 weeks, 3.75 g | 3 weeks, 7.5 g | 1.759 | 0.157 | -1.372 | 0.340 |
|  | 3 weeks, 3.75 g | 6 weeks, 7.5 g | 1.766 | 0.194 | 0.709 | 0.478 |
|  | 3 weeks, 7.5 g | 6 weeks, 7.5 g | -0.265 | 0.396 | 2.243 | 0.062 |
| **Cu** | no ash | 3 weeks, 3.75 g | -1.015 | 0.620 | -1.131 | 0.516 |
|  | no ash | 3 weeks, 7.5 g | -1.280 | 0.601 | 0.978 | 0.328 |
|  | no ash | 6 weeks, 7.5 g | -0.216 | 0.414 | -1.063 | 0.432 |
|  | 3 weeks, 3.75 g | 3 weeks, 7.5 g | 0.229 | 0.819 | -1.886 | 0.148 |
|  | 3 weeks, 3.75 g | 6 weeks, 7.5 g | -0.839 | 0.602 | -0.211 | 0.417 |
|  | 3 weeks, 7.5 g | 6 weeks, 7.5 g | -1.104 | 0.674 | 1.898 | 0.173 |
| **Zn** | no ash | 3 weeks, 3.75 g | 0.839 | 1.000 | 1.112 | 0.266 |
|  | no ash | 3 weeks, 7.5 g | -0.132 | 0.447 | 1.591 | 0.223 |
|  | no ash | 6 weeks, 7.5 g | 0.541 | 0.883 | -1.129 | 0.388 |
|  | 3 weeks, 3.75 g | 3 weeks, 7.5 g | 0.841 | 1.000 | -0.429 | 0.334 |
|  | 3 weeks, 3.75 g | 6 weeks, 7.5 g | 0.397 | 0.691 | 2.090 | 0.092 |
|  | 3 weeks, 7.5 g | 6 weeks, 7.5 g | -0.574 | 1.000 | 2.569 | **0.031** |
| **δ^56^Fe** | no ash | 3 weeks, 3.75 g | -1.854 | 0.127 | 1.501 | 0.267 |
|  | no ash | 3 weeks, 7.5 g | -1.766 | 0.116 | 2.119 | 0.102 |
|  | no ash | 6 weeks, 7.5 g | 0.433 | 0.665 | 1.839 | 0.165 |
|  | 3 weeks, 3.75 g | 3 weeks, 7.5 g | -0.076 | 0.470 | -0.535 | 0.592 |
|  | 3 weeks, 3.75 g | 6 weeks, 7.5 g | -2.208 | 0.082 | 0.000 | 0.500 |
|  | 3 weeks, 7.5 g | 6 weeks, 7.5 g | -2.119 | 0.085 | 0.618 | 0.805 |
| **δ^65^Cu** | no ash | 3 weeks, 3.75 g | -3.109 | **0.006** | 0.177 | 0.860 |
|  | no ash | 3 weeks, 7.5 g | -2.296 | 0.054 | -0.442 | 0.988 |
|  | no ash | 6 weeks, 7.5 g | -2.115 | 0.069 | -0.649 | 1.000 |
|  | 3 weeks, 3.75 g | 3 weeks, 7.5 g | -0.728 | 0.467 | 0.535 | 1.000 |
|  | 3 weeks, 3.75 g | 6 weeks, 7.5 g | -1.400 | 0.242 | 0.706 | 1.000 |
|  | 3 weeks, 7.5 g | 6 weeks, 7.5 g | -0.560 | 0.288 | 0.088 | 0.465 |
| **δ^66^Zn** | no ash | 3 weeks, 3.75 g | 1.015 | 0.620 | -1.545 | 0.306 |
|  | no ash | 3 weeks, 7.5 g | 2.163 | 0.092 | 0.221 | 0.413 |
|  | no ash | 6 weeks, 7.5 g | 1.947 | 0.129 | 1.136 | 0.384 |
|  | 3 weeks, 3.75 g | 3 weeks, 7.5 g | -0.994 | 0.480 | -1.529 | 0.252 |
|  | 3 weeks, 3.75 g | 6 weeks, 7.5 g | -0.574 | 0.566 | -2.472 | **0.040** |
|  | 3 weeks, 7.5 g | 6 weeks, 7.5 g | 0.574 | 0.283 | -0.706 | 0.480 |

Table S10: Correlation analysis of δ^65^Cu in the soft tissue of the coral *Stylophora pistillata* with various photosynthetic parameters (Photosynthesis and respiration rates and Φ_PSII_) and metal concentrations (Cu and Zn).

The sample size comprises the means of individual observations per aquaria, except for the metal concentration (Cu and Zn). Statistically significant correlations exist (**bold**) when p ≤ 0.05.

| **Measurement** | | **Sample size** | **Pearson R^2^** | **p-value** |
| --- | --- | --- | --- | --- |
| *Measurement 1* | *Measurement 2* |  |  |  |
| δ^65^Cu_Coral host_ | Φ_PSII_ | 6 | 0.720 | **0.033** |
| δ^65^Cu_Coral host_ | Photosynthesis rate  normalized per surface area | 4 | 0.978 | **0 .012** |
| δ^65^Cu_Coral host_ | Respiration rate  normalized per surface area | 4 | 0.650 | 0.194 |
| δ^65^Cu_Coral host_ | Photosynthesis rate  normalized per symbiont cell | 4 | 0.983 | **0.009** |
| δ^65^Cu_Coral host_ | Respiration rate  normalized per symbiont cell | 4 | 0.969 | **0.016** |
| δ^65^Cu_Symbions_ | Photosynthesis rate  normalized per surface area | 4 | 0.001 | 0.975 |
| δ^65^Cu_Symbions_ | Respiration rate  normalized per surface area | 4 | 0.244 | 0.506 |
| δ^65^Cu_Symbions_ | Photosynthesis rate  normalized per symbiont cell | 4 | 1.97E-04 | 0.986 |
| δ^65^Cu_Symbions_ | Respiration rate  normalized per symbiont cell | 4 | 2.52E-05 | 0.995 |
| δ^65^Cu_Coral host_ | Cu/Zn_Coral host_ | 4 | 0.616 | 0.215 |
| Cu_Coral host_ | Zn_Coral host_ | 18 | 0.710 | **1.10E-05** |
| Cu_Symbionts_ | Zn_Symbionts_ | 16 | 0.652 | **1.56E-04** |

Table S11: Correlation analysis of δ^65^Cu_Coral host_ in the coral *Stylophora pistillata* with the rapid light curves (RLC) derived photochemical parameters (F_v_/F_m_, rETR and NPQ) across 11 light intensities (expressed as PAR in µmol photons/m^2^/s).

The sample size in (a) comprises the means of individual observations (presented in b). Statistically significant correlations exist (**bold**) when p ≤ 0.05.

| **a** |  |  | |  | |  | |
| --- | --- | --- | --- | --- | --- | --- | --- |
| **PAR** | **Sample size** | **F_v_/F_m_** | | **ETR** | | **NPQ** | |
|  |  | *Pearson R^2^* | *p-value* | *Pearson R^2^* | *p-value* | *Pearson R^2^* | *p-value* |
| 0 | 4 | 0.751 | 0.134 | - | - | - | - |
| 10 | 4 | 0.786 | 0.114 | 0.796 | 0 .108 | 0.136 | 0.632 |
| 17 | 4 | 0.787 | 0.113 | 0.789 | 0.112 | 0.621 | 0.212 |
| 26 | 4 | 0.793 | 0.110 | 0.790 | 0.112 | 0.943 | **0.029** |
| 41 | 4 | 0.787 | 0.114 | 0.790 | 0.112 | 0.890 | 0.057 |
| 74 | 4 | 0.771 | 0.122 | 0.769 | 0.124 | 0.863 | 0.071 |
| 130 | 4 | 0.812 | 0.099 | 0.812 | 0.099 | 0.843 | 0.082 |
| 220 | 4 | 0.784 | 0.115 | 0.785 | 0.115 | 0.833 | 0.088 |
| 664 | 4 | 0.019 | 0.862 | 0.018 | 0.866 | 0.837 | 0.085 |
| 1032 | 4 | 0.384 | 0.381 | 0.394 | 0.372 | 0.841 | 0.083 |
| 1960 | 4 | 0.528 | 0.273 | 0.530 | 0.272 | 0.843 | 0.082 |
| **b** |  |  |  |  |  |  |  |
| **PAR** | **Sample size** | **F_v_/F_m_** | | **ETR** | | **NPQ** | |
|  |  | *Pearson R^2^* | *p-value* | *Pearson R^2^* | *p-value* | *Pearson R^2^* | *p-value* |
| 0 | 12 | 0.520 | **0.008** | - | - | - | - |
| 10 | 12 | 0.501 | **0.010** | 0.503 | **0.010** | 0.206 | 0.139 |
| 17 | 12 | 0.506 | **0.010** | 0.509 | **0 .009** | 0.004 | 0.844 |
| 26 | 12 | 0.507 | **0.009** | 0.495 | **0.011** | 0.233 | 0.112 |
| 41 | 12 | 0.507 | **0.010** | 0.508 | **0.009** | 0.362 | **0.038** |
| 74 | 12 | 0.470 | **0.014** | 0.467 | **0.014** | 0.409 | **0.025** |
| 130 | 12 | 0.480 | **0.012** | 0.482 | **0 .012** | 0.428 | **0.021** |
| 220 | 12 | 0.396 | **0.028** | 0.395 | **0.029** | 0.451 | **0.017** |
| 664 | 12 | 0.009 | 0.767 | 0.008 | 0.7810 | 0.477 | **0.013** |
| 1032 | 12 | 0.186 | 0.162 | 0.193 | 0.1520 | 0.494 | **0.011** |
| 1960 | 12 | 0.172 | 0.180 | 0.172 | 0.180 | 0.504 | **0.010** |
